# Supplementary material for: Triadic embeddedness structure in family networks predicts mobile communication response to a sudden natural disaster
Source: Nat Commun. 2021 Jul 13;12:4286. doi: 10.1038/s41467-021-24606-7 (PMC8277904; doi:10.1038/s41467-021-24606-7)
Supplement: Supplementary file 1 — Supplementary Information [file 41467_2021_24606_MOESM1_ESM.pdf]

Supplementary Information

Triadic Embeddedness Structure in Family Networks Predicts Mobile Communication Response to a Sudden Natural Disaster

Jayson S. Jia[1↑*], Yiwei Li[2↑], Xin Lu[3,4], Yijian Ning[5,6], Nicholas A. Christakis[7], Jianmin Jia[8,9↑*]

[1] Faculty of Business and Economics, The University of Hong Kong, Hong Kong SAR, China.
[2] Department of Marketing & International Business, Faculty of Business, Lingnan University, Hong Kong SAR, China.
[3] College of Systems Engineering, National University of Defense Technology, Changsha, China
[4] School of Business, Central South University, Changsha, China
[5] School of Economics and Management, Southwest Jiaotong University, Chengdu, China.
[6] Service Science and Innovation Key Laboratory of Sichuan Province, Chengdu, China.
[7] Yale Institute for Network Science, Yale University, New Haven, CT, U.S.A.
[8] Shenzhen Finance Institute, School of Management and Economics, The Chinese University of Hong Kong, Shenzhen, China.
[9] Shenzhen Research Institute of Big Data, Shenzhen, China
[↑] Contributed equally

[*]Corresponding authors. Email: jmjia@cuhk.edu.cn (JMJ); jjia@hku.hk (JSJ)

Table of Contents

Earthquake and Background 4
WeChat Robustness Check for Ya'an voice call data 5
The stability of voice calls and SMS usage over time. 6
Triadic family embeddedness structure 7
The embeddedness structures for 3-person families. 7
Telecom network characteristics for each triadic embeddedness structure type. 8
Descriptive statistics for each structure of embeddedness 8
Family Survey 8
Survey response frequency by embeddedness structural types 9
Family roles within different embeddedness structural types 10
Stability of social networks before and after the earthquake 10
Overlap in number of common friends of family for before and after earthquake. 10
Number of unique embedded and non-embedded friends before and after the earthquake. 11
Mean number of common and normal friends. 11
Additional Figures for Model-free Analyses 12
Effects of strength versus structure of family embeddedness. 12
Effect of tie strength and embeddedness structure on likelihood and proportion of calls to friends. 13
Effect of embeddedness structure and degree centrality. 14
Robustness Checks for Models 1-2 16
First inbound call robustness checks 16
Predicting probability that first *inbound* call is from a non-family tie 16
Predicting latency until first *inbound* call to non-family plan member 17
Model diagnostics for Bayesian Estimation of Model 2 18
Roaming only robustness checks 19
Panel models with earthquake shock 20
Transition matrix of the three triads before and after the earthquake 20
Average of network outcome variables in experiment and control groups across time 21
Individual-Fixed Effects Robustness checks. 23
Predicting reciprocity (Model 3) with individual fixed effects 23
Predicting degree centrality (Model 4) with individual fixed effects 24
Embeddedness strength manipulation checks 25
Predicting initial outbound calls with embeddedness strength 25
Predicting first outbound call latency with embeddedness strength 26
Predicting reciprocity with embeddedness strength 27
Predicting degree centrality with embeddedness strength 28
Variance of Family Embeddedness 29
Stability of mean and variance of embeddedness structure. 30

Predicting initial outbound calls with variance of family embeddedness 31
Predicting first outbound call latency with variance of family embeddedness 32
Predicting reciprocity with variance of family embeddedness 33
Predicting degree centrality with variance of family embeddedness 34
Miscellaneous Robustness Checks 35
Robustness check for Model 1 using 4 person families 35
Robustness check for Model 2 using 4 person families 36
Robustness check comparing family Type 1 vs. 2 for Model 1 37
Robustness check comparing family Type 1 vs. 2 for Model 2 38
Triadic motifs and changes in intra-family communications.. 41
Relative change of motifs before and after earthquake for different embeddedness structures.45
Counts for types of motifs with following social network properties 46
Visualization of motif transition matrix before and after earthquake....... **Error! Bookmark not defined.**
Macro-network effects and giant connected component graphs... 47
Intra vs. extra- family communications after earthquake. 47
Communications within and between families before the earthquake. 48
Communications within and between families after the earthquake. 49
A cluster with relatively fewer inter-family communications 50
A cluster with relatively more inter-family communications 51
Supplementary References... 52

Earthquake and Background

The Yaan earthquake occurred at 08:02 (Beijing Time, UTC +8:00) on April 20 (Saturday), 2013, with the epicenter located in Lushan County, Yaan, Sichuan, about 116 km from Chengdu, Sichuan (in southwest China). The China Earthquake Data Center placed the magnitude of the earthquake at Ms 7.0.

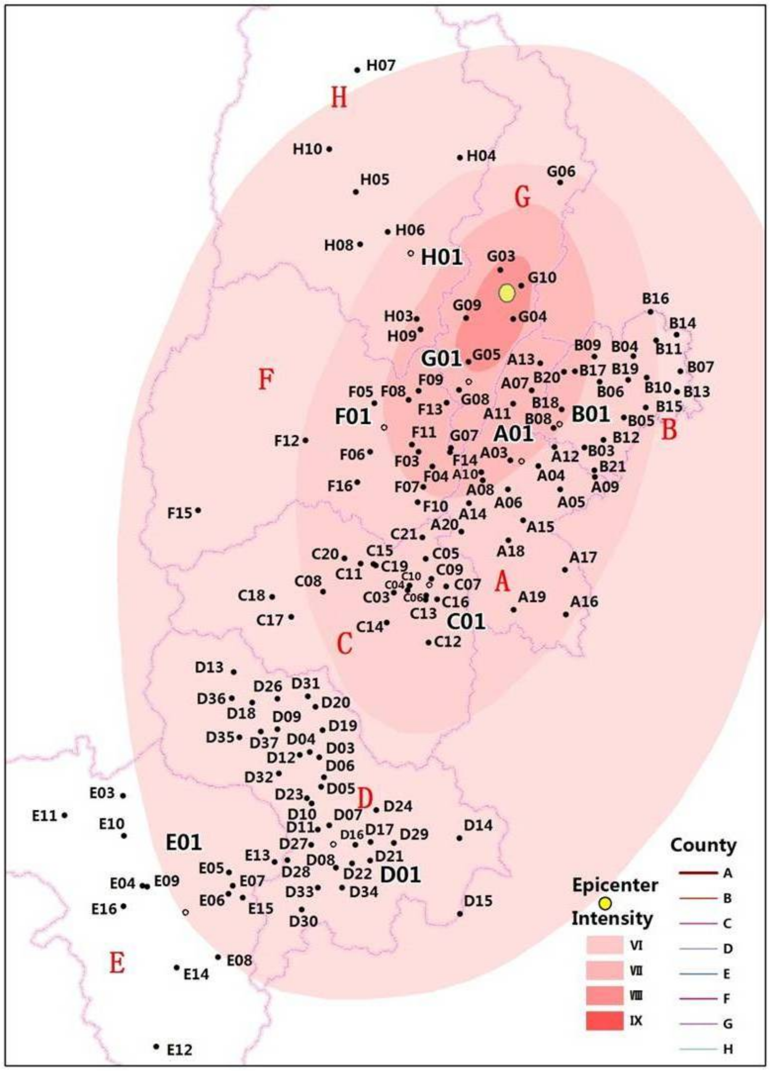

Supplementary Figure 1. Geographic distribution of earthquake intensity in Ya'an prefecture. The region contains one main city (A) and seven counties (B-H); each of them has one city/county seat and many towns/villages (A01, A02…).

Earthquake intensity (Typical Maximum Modified Mercalli Intensity) is a categorical measure reflecting physical damage sustained after the earthquake. Notably, it is different from the Richter scale or peak ground acceleration which measure amount of energy released. According to the US geological survey, 4.0-4.9 on the Richter scale roughly corresponds to IV-V

intensity, 5.0-5.9 to VI-VII intensity, 6.0-6.9 to VII-IX intensity, 7.0 and higher to VIII or higher. Ya'an regions suffered intensities ranging from V to IX:

Typical Maximum Modified Mercalli Intensity (from US Geological Survey):

V. Felt by nearly everyone; many awakened. Some dishes, windows broken. Unstable objects overturned. Pendulum clocks may stop.

VI. Felt by all, many frightened. Some heavy furniture moved; a few instances of fallen plaster. Damage slight.

VII. Damage negligible in buildings of good design and construction; slight to moderate in well-built ordinary structures; considerable damage in poorly built or badly designed structures; some chimneys broken.

VIII. Damage slight in specially designed structures; considerable damage in ordinary substantial buildings with partial collapse. Damage great in poorly built structures. Fall of chimneys, factory stacks, columns, monuments, walls. Heavy furniture overturned.

IX. Damage considerable in specially designed structures; well-designed frame structures thrown out of plumb. Damage great in substantial buildings, with partial collapse. Buildings shifted off foundations.

Our analyses separates the effect of the earthquake into two levels: Magnitude VII and below, and magnitude VIII and above, with the logic that everyone in the dataset experienced a major earthquake, and that the primary difference in impact was whether there was (greater than negligible) physical damage or not (i.e., magnitude VIII and above).

WeChat Robustness Check for Ya'an voice call data

In the main text, we mention that call data is suitable for capturing people's social networks, and that mobile phone call data has been a commonly used form of data for network research (e.g., Barabási 2005, Eagle, Pentland, Lazer 2009, Jo et al. 2014, Lu, Bengtsson, Holme 2012, Palchykov et al. 2012, Onnela et al. 2007, Saramäkia et al. 2014). However, one might wonder if the rapid adoption of WeChat across China affected the internal validity of our operatoinalization, i.e., whether there was a major substitution effect for calls during the time of our study: March to June, 2013.

Our data suggests this was not the case; we did not observe a decline in voice call usage between January 2013 and July 2016 in Yaan. Call duration, for both in- and out-bound calls were very stable over time (Supplementary Fig. 2a); call frequency did not decline over time either (Supplementary Fig. 2a). However, we observed a significant declining trend in the frequency of outbound text messages (Supplementary Fig. 2b). Overall, we only find evidence of a WeChat substitution effect for outbound text messages but not for voice calls between January 2013 and July 2016.

A

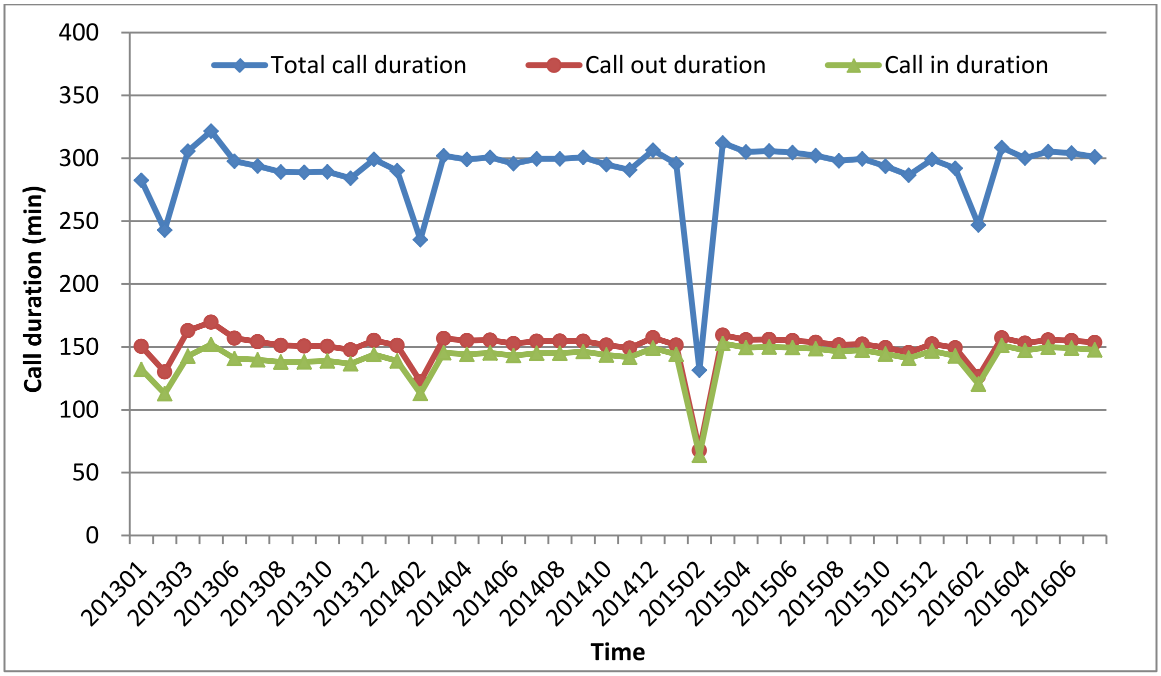

B

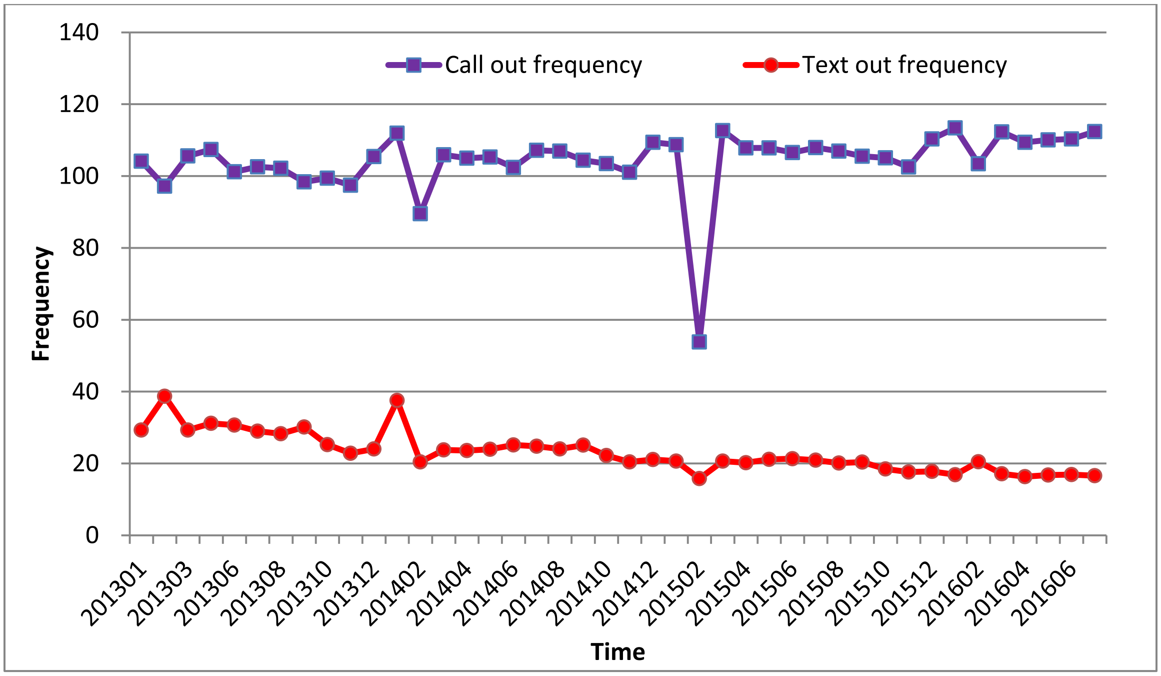

Supplementary Figure 2. The stability of call duration over time (A) and the trend of call out and text out message usage over time (B).

The seasonal dips in communications duration and frequency correspond to the Lunar New Year holidays, when almost everyone is off work and visiting family. It is possible that WeChat had a significant effect on calls and texts during the Lunar New Year holidays in 2015, which was almost two years later after the earthquake.

According to Tencent's annual report, WeChat had 194 million of monthly active users by the end of Quarter 1 of 2013. WeChat started to gain popular among younger demographics in major urban areas in late 2013; during this time, it was not yet popular in rural or small-town geographic locales such as Ya'an, particularly among older demographic segments. Based on our data, 23.65% of users in our dataset had the WeChat app installed, but only 15.93% used WeChat more than 5 times monthly before the earthquake. The Pearson correlation between WeChat and outbound calls is 0.148 ($p > .001$) before the earthquake and 0.165 ($p > .001$) after the earthquake, which shows a complementarity rather than substitution effect. During the study period, WeChat did not yet have good voice call function (WeChat only introduced its VOIP call service in 2016). Overall, it seems unlikely that WeChat was the most popular first means of communications during the Ya'an earthquake. We also found people generally used voice calls rather than SMS to communicate with family members immediately after the earthquake.

Triadic family embeddedness structure

Our primary analyses focuses on 3 person family structures for empirical tractability, and because triads are significant for being the smallest form of a social network. Such triadic structures frequently appear in kinship systems (Sweetser 1967), especially in nuclear families (Freilich 1964). However, it should be possible to extend the construct of embeddedness structure to family structures with more nodes. Indeed, as a robustness check of family embeddedness structure, we extend analyses to four-person family plans in Supplementary Tables S19-20.

There are six possible types of embeddedness structures for 3-person families if we differentiate ego from alters:

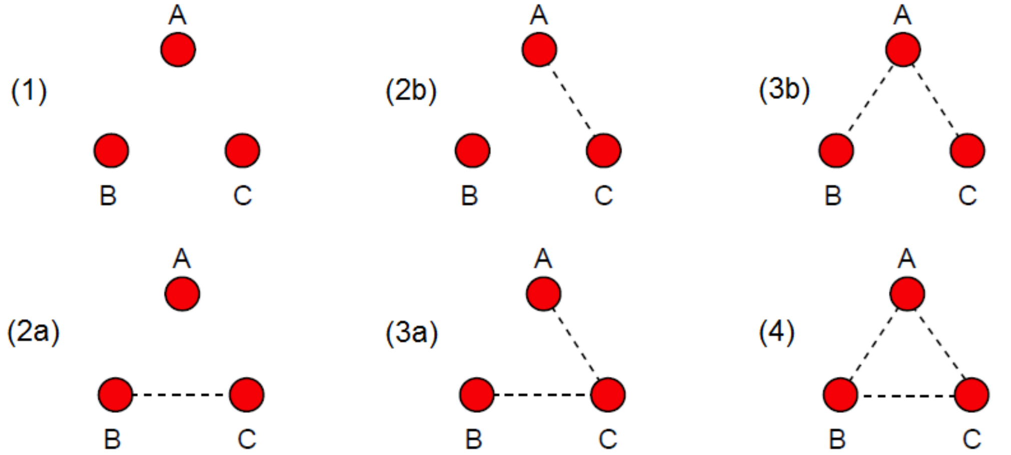

Supplementary Figure 3. The embeddedness structures for 3-person families. The ego is node A, and the two nodes B and C are exchangeable; dotted-lines represent embeddedness relations (i.e., the corresponding two nodes have at least one shared friend). A triadic family relation can be fully embedded (type 4) or completely unembedded (type 1) even when the

family members have direct communications. Below, we tabulate the relative frequencies of all embeddedness structures based on telecom data from the four weeks before the earthquake. Note that in our primary analysis (Fig. 1C), we do not distinguish between (2a) and (2b) or (3a) and (3b).

Supplementary Table 1. Telecom network characteristics for each triadic embeddedness structure type.

Embeddedness Structure type	Relative frequency %	Degree centrality	Total call frequency	Total call frequency to family	Total text frequency	Total internet frequency
1	7.31	39.0	212	3.10	65.0	179
2a	1.39	10.1	53.0	7.58	15.2	67.5
2b	24.1	39.9	222	26.5	58.1	162
3a	4.83	24.7	129	20.8	37.7	106
3b	1.89	36.3	192	25.4	62.6	135
4	60.4	34.7	196	40.7	47.4	141

Supplementary Table 2. Descriptive statistics for each structure of embeddedness

Structural type of triadic embeddedness	Likelihood first outbound call is to family		Proportion of calls to family during the first week		Latency of the first outbound call after the earthquake		
	Mean	S.E.	Mean	S.E.	Median	Mean	S.E.
1	0.08	0.01	0.06	<0.01	4.18	26.09	1.56
2a	0.22	0.02	0.19	0.02	12.29	84.34	6.89
2b	0.21	<0.01	0.19	<0.01	3.45	19.44	0.67
3a	0.29	0.01	0.28	0.01	4.62	40.97	2.61
3b	0.27	0.02	0.21	0.01	3.55	14.36	1.66
4	0.34	<0.01	0.32	<0.01	3.28	18.19	0.40

Family Survey

Previous research typically relies exclusively on surveys to identify relationships between families, which have the typical limitations of self-report (reporting inaccuracies, memory biases, self-presentation biases, etc.). In our dataset, family plan membership is a more objective proxy for family membership since the telecom company is obligated to check applicants' identity cards upon registration for family plans. Furthermore, families are incentivized to sign up for family plans since they provide price discounts.

To cross-validate that family plan members were indeed kin, we conducted an independent phone survey of all numbers in 2000 randomly drawn family plan subscriptions, which were active at the time of the earthquake, from the dataset two years after the earthquake.

We defined active plans as families with at least 10 intra-family communications in the first 10 days of the earthquake.

We called and surveyed the 6000 phone numbers individually from the 2000 families with 3 persons (response rate = 45.7%). We first asked a few questions unrelated to this study including customer and service satisfaction and extent of earthquake damage. We then asked participants to identify the family roles (e.g., mother, paternal grandfather, etc.) of the other users in their family plan.

Survey results showed that 40.8% of family plan members were parents, 31.1% were couples, 22.6% were children, 1.71% were grandparents, and 3.83% other kinship ties (e.g., siblings); average family size was 3.70; 72.5% of the family plan members lived together. Note that individuals may have multiple roles within family plans, for example, in a family plan of 3, subscriber 1 may be the husband of subscriber 2 and son of subscriber 3.

Although most respondents were members of a nuclear family, it should be noted that our research hypotheses are unaffected by the degree of kinship, but rather assume that family plan membership is socially significant. Indeed, it is economically meaningful that the individuals signed up for family plans together, and that the primary account holder incurred the burden (or at least risk) of paying for others' mobile telecommunications services in a relatively low-income region of China. Similarly, it is socially meaningful (and akin to "revealed closeness") that an individual chooses to pay for the mobile phone bill of some kin but not others. We also observed that the response rate was far higher for fully embedded families (which account for 93.6% of survey respondents but 60.4% of families in the telecom dataset), and almost zero for fully unembedded families (7.4% of the dataset). This selection effect may have been the result of our initial sampling rule (of surveying families that had active communications between all dyads). Another possibility is that fully unembedded families were more likely to churn from the family plan or telecom service (since the survey was conducted 2 years after the earthquake, some families may have left the carrier). Yet another possibility is that the response rate to our survey was correlated with the general communications responsiveness of families (which is lower for completely unembedded families).

Supplementary Table 3. Survey response frequency by embeddedness structural types

Structure Type	Frequency of responses	Response frequency as %
1	3	0.05
2	109	1.8
3	266	4.4
4	5614	93.6

We further considered if family role has any relationship with family structure types. We provide the relative frequencies of family types of our survey in the interests of data transparency, but urge extreme caution in over interpreting the data in light of the response bias. Within surveyed families, at least, there were no significant differences in family roles between different structure types.

Supplementary Table 4. Family roles within different embeddedness structural types

Structure Type	Parents (%)	Children (%)	Grandparents (%)	Cohabitating (%)	Number cohabitating (mean)
2 (1 embedded link)	35.8	8.3	-	22.9	4
3 (2 embedded links)	30.1	9.4	1.5	18.0	4
4 (fully embedded)	32.9	10.4	1.4	18.1	4

Stability of social networks before and after the earthquake

Here we explore whether the composition of individuals' social network structures changes before or after the earthquake (which we also revisit in the panel model analyses later). As discussed in the main text, the embeddedness structure of most families remained stable. Average embeddedness structure (as measured by number of family dyads that are embedded) remains stable across geographical locations after the earthquake (Supplementary Fig. 4).

We explored whether the similarity of the pre- and post- earthquake social networks, particularly in relation to shared social resources, i.e., friends that are embedded or not embedded into the family, and the proportion of the two. We first created a metric measuring the two types of extra-familial relationships.

Let the set of common friends with at least two family members be A and B for before and after earthquake, and let the set of intersect (A, B) be C, then for either common friends or ordinary friends (i.e., those who have interacted with at least a family member), the ratio of overlapped friends is calculated by $r = C/\min(|A|, |B|)$.

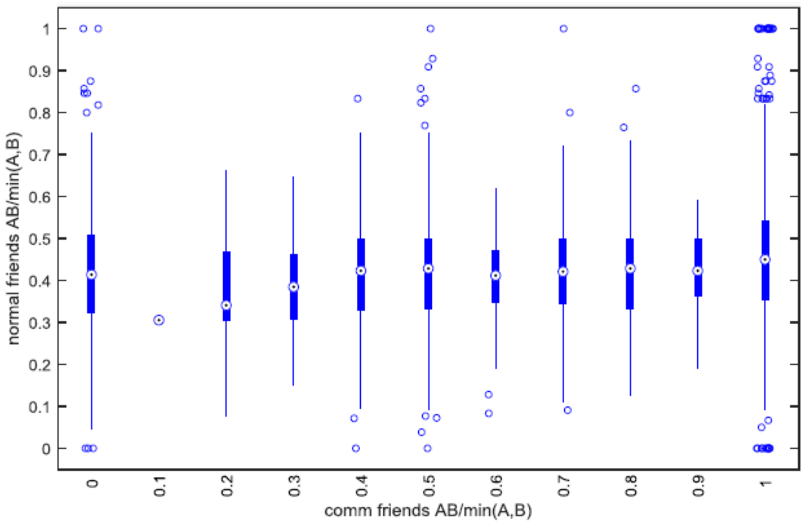

Supplementary Figure 4. Overlap in number of common friends and normal friends of family.

In Supplementary Figure 4, we group the N = 11,855 three-person family plans according to the deciles of the ratio for common friends' overlap and the deciles of the ratio of normal friends' overlap. We can see that the ratio of common friends (embeddedness) is independent of normal friends , and lies in the region of 0.3~0.5.

We then explored if the number of common or normal friends changed after the earthquake for the N = 35,565 users from the 11,855 three-person family plans.

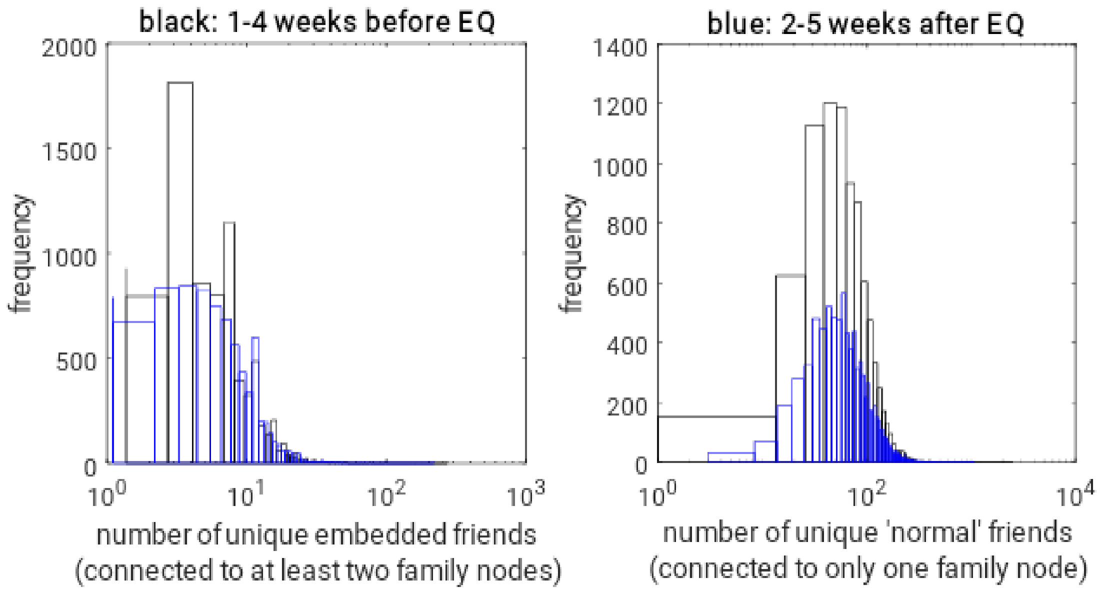

Supplementary Figure 5. Number of unique embedded and non-embedded friends before and after the earthquake. Supplementary Figure 5 shows the frequency distribution of unique embedded and unique 'normal' friends before and after the earthquake.

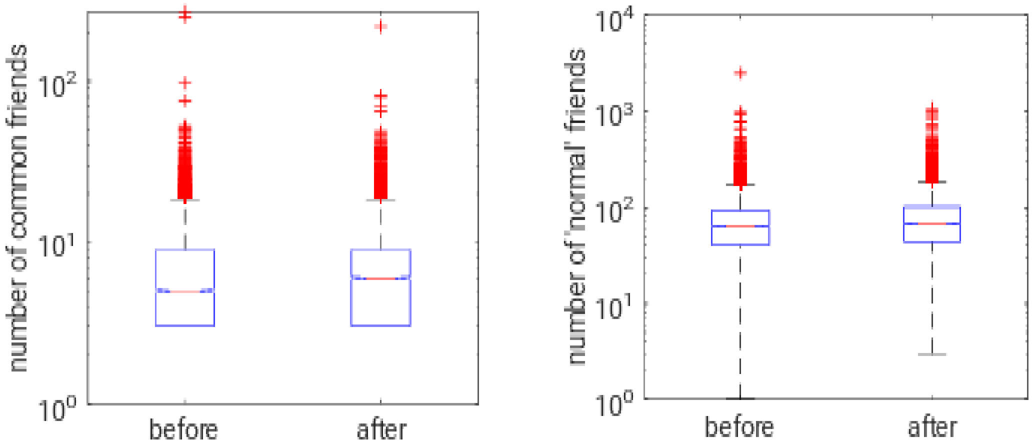

Supplementary Figure 6. Mean number of common (left) and normal (right) friends. Supplementary Figure 6 shows that the number of common and normal friends is similar both before and after the earthquake. Error bars denote 95% confidence interval.

Additional Figures for Model-free Analyses

The figures below supplement the model free analyses and Figure 2 of the main text (N = 35,565 users from 11,855 three-person family plans).

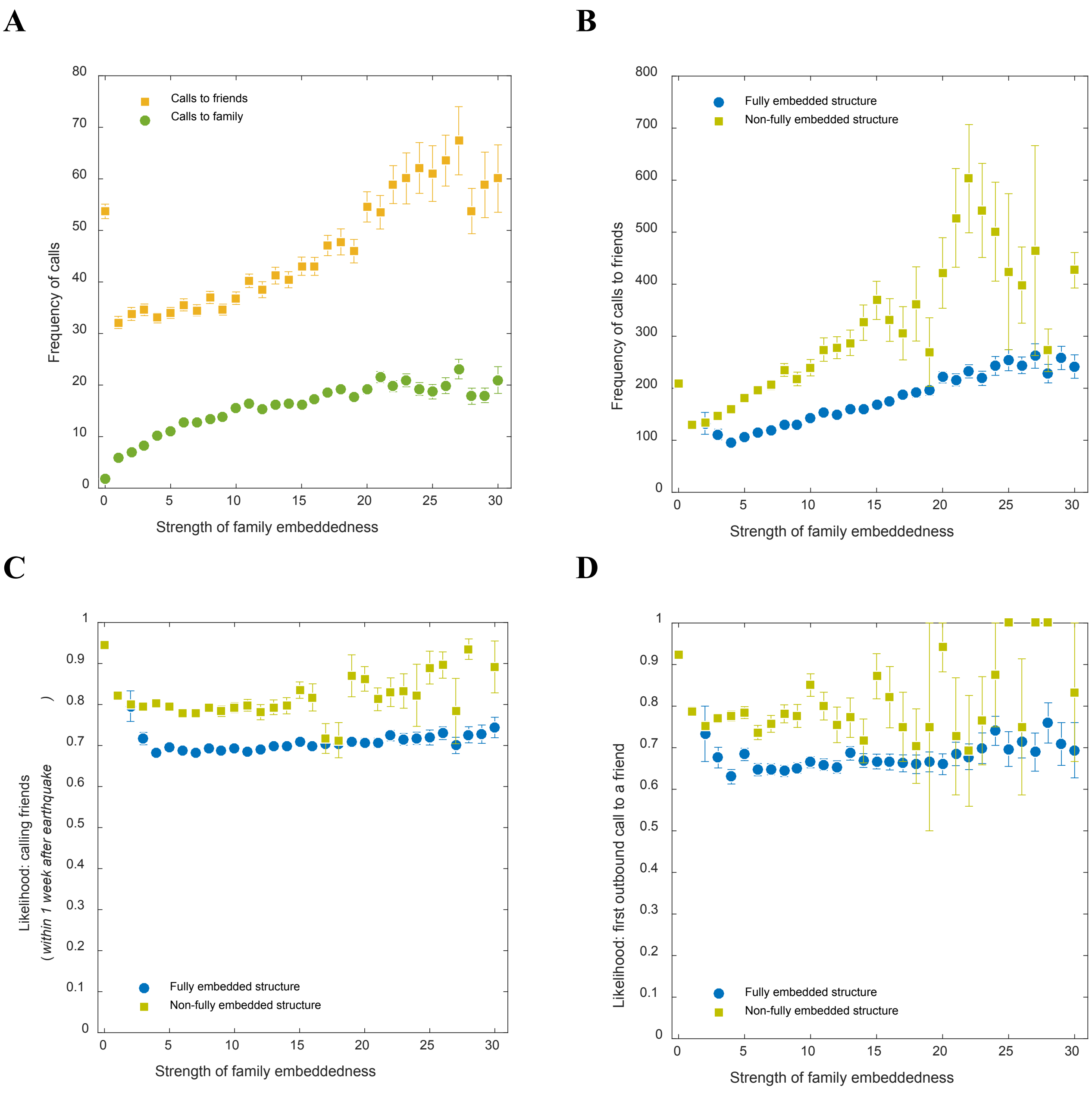

Supplementary Figure 7. Effects of strength versus structure of family embeddedness. The effects of the strength and structure of family embeddedness; the former is measured by total number of common friends between family members; the latter is represented by dummies for fully embedded structure (type 6) and non-fully embedded ones (types 1-5). (**A**) Frequency of calls to friends and family both increase with strength of family embeddedness, however the former (latter) has a decreasing (increasing) rate of change. (**B**) Overall, there are significantly

fewer calls to friends in fully embedded families than in non-fully embedded families. **C** and **D** show that strength of family embeddedness has little effect, while structure of family embeddedness has a significant effect, on who the ego calls after the earthquake (friends vs. family). Egos in fully embedded families, relative to egos in non-fully embedded families, (**C**) make a smaller proportion of their total post-earthquake calls to friends (Pr = .697 vs. .826, $p < .001$), or (**D**) and are less likely to first call a non-family member after the earthquake (Pr = .664 vs. .800, $p < .001$). N = 35,565 users. Error bars denote 95% confidence interval.

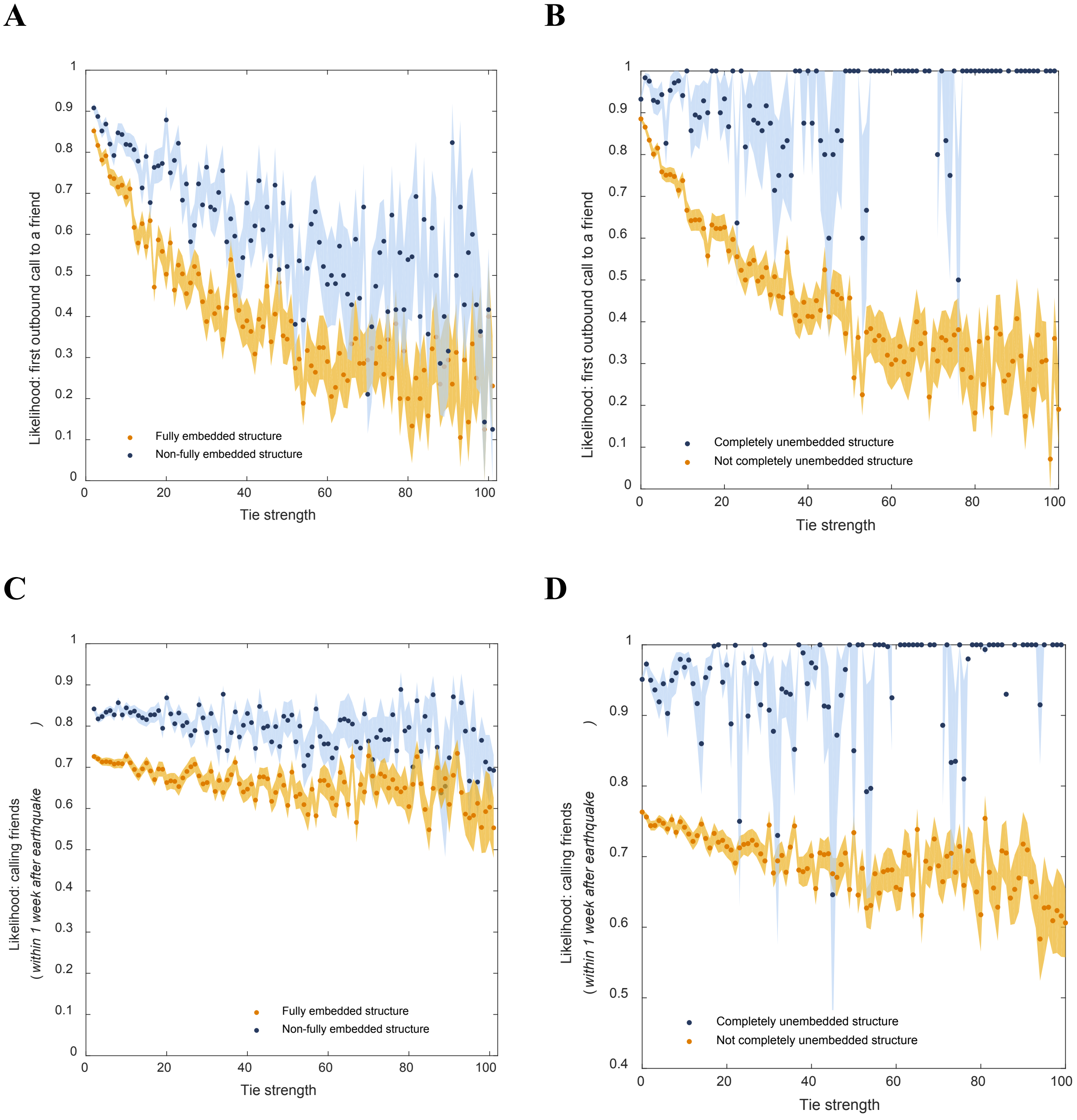

Supplementary Figure 8. Effect of tie strength and embeddedness structure on likelihood and proportion of calls to friends. Controlling for tie strength, egos are significantly more

likely to first call a friend after the earthquake when they are in (**A**) fully embedded as compared to non-fully embedded family structures, and (**B**) when they are in completely unembedded as compared to non-completely unembedded family structures. Analogously, egos direct a higher proportion of calls to friends (as opposed to family) when they are in (**C**) fully embedded as compared to non-fully embedded family structures, and (**D**) when they are in completely unembedded as compared to non-completely unembedded family structures. N = 35,565 users, Error bands denote 95% CI.

A

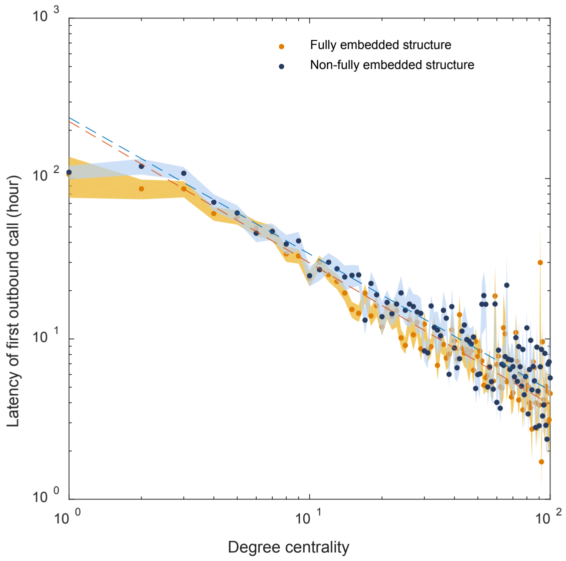

B

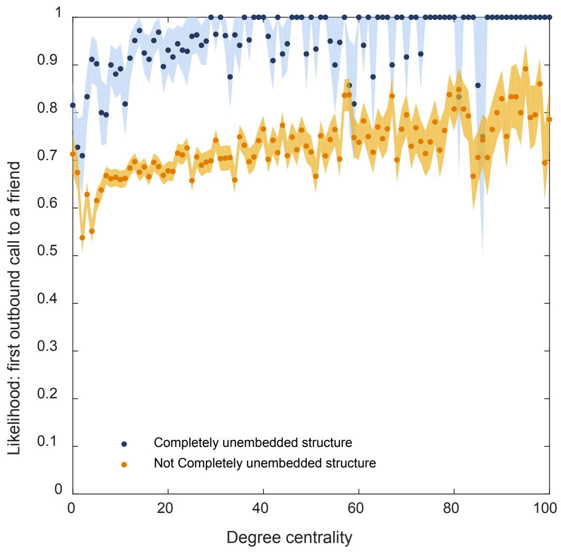

C

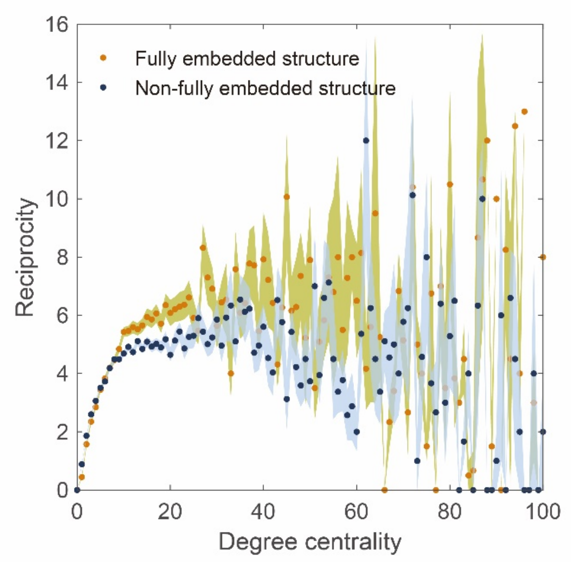

Supplementary Figure 9. Effect of embeddedness structure and degree centrality. (**A**) Both degree centrality and triadic embeddedness structure have an effect on the latency of the first

outbound call. (**B**) Egos were more likely to first call a friend when they are in completely unembedded versus non-completely unembedded family structures (0.924 vs. 0.702, $p < .001$). (**C**) Egos are more reciprocal when they are in completely embedded versus non-fully embedded structures (5.584 vs. 4.392, $p < .001$). N = 35,565 users. Error bands denote 95% CI.

Overall, the behavioural outcomes of embeddedness structure reflected their conceptual meaning; fully embedded families were the most close-knit, since every member shared part of their social network with every other family member. However, completely unembedded families, where no family member shared friends, were the least close-knit. Their relative closeness is behaviourally reflected in whether they chose to call each other first, and by their relative urgency in activating (non-family) friendship networks.

Robustness Checks for Models 1-2

First inbound call robustness checks

Here we conduct the analogous analysis as Table 1a in the main text, but for first inbound calls. We obtain analogous results, namely a positive interaction between fully unembedded family structures and earthquake intensity on likelihood of receiving a call from a non-family member first.

Supplementary Table 5. Predicting probability that first *inbound* call is from a non-family tie

DV = *p*(first inbound call is from non-family plan member)	Coef.	S.E.	*z*	*p*>|*z*|	
(1) Ego and family all unembedded	0.624	0.069	9.04	<0.001	***
(2) Ego and family all embedded	-0.320	0.027	-11.72	<0.001	***
Earthquake intensity group (1 = severe)	0.181	0.036	5.08	<0.001	***
(1)*earthquake intensity group	0.296	0.113	2.61	0.009	**
(2)*earthquake intensity group	-0.022	0.043	-0.52	0.606	
ln(Degree centrality of ego)	0.349	0.024	14.56	<0.001	***
ln(Total call frequency of ego)	-0.165	0.018	-9.13	<0.001	***
ln(Total text frequency of ego)	0.037	0.007	5.42	<0.001	***
ln(Internet usage frequency of ego)	-0.008	0.006	-1.32	0.186	
ln(Phone retail price in Yuan)	-0.001	0.013	-0.11	0.912	
ln(Total WeChat frequency)	0.016	0.007	2.23	0.026	*
ln(Total frequency of other instant messaging)	-0.010	0.006	-1.63	0.104	
Roaming dummy (1 = traveling outside of prefecture)	-0.389	0.027	-14.42	<0.001	***
Rural dummy (1 = rural)	-0.105	0.021	-5.01	<0.001	***
Damage dummy (1 = cell towers damaged in neighbourhood)	0.253	0.071	3.55	<0.001	***
Prior intra-family outbound call share	-0.970	0.072	-13.57	<0.001	***
(Intercept)	0.604	0.092	6.54	<0.001	***

Notes. Number of obs. = 26,394; Controlled for random effects of families; The *p*-values are obtained from a two-sided *z*-test; $^{***}p < .001$, $^{**}p < .01$, $^{*}p < .05$, $^{\bullet}p < .1$

Here we conduct the same analysis as Model 2 (Table 1b in Main Text), but for temporal latency of first inbound calls. We obtain analogous results; a negative interaction between fully unembedded family structure and earthquake intensity, as well as a positive interaction effect between fully embedded family structure and earthquake intensity on latency until first call from a non-family member.

Supplementary Table 6. Predicting latency until first *inbound* call to non-family plan member

DV = Hours until first inbound call from non-family plan member	Coef.	S.D.	Lower 2.5% CI	Upper 97.5% CI	N_eff	Rhat
(1) Ego and family all unembedded	-25.047	4.869	-34.587	-15.353	17655	1.000
(2) Ego and family all embedded	58.525	4.717	49.391	67.648	18798	1.000
Earthquake intensity group (1 = severe)	-29.401	4.709	-38.704	-20.169	20109	1.000
(1)*earthquake intensity group	-14.418	5.111	-24.578	-4.373	16547	1.000
(2)*earthquake intensity group	8.126	3.899	0.347	15.818	15859	1.000
ln(Degree centrality of ego)	-69.371	4.281	-77.578	-60.888	13873	1.000
ln(Total call frequency of ego)	-36.844	3.737	-44.169	-29.452	12471	1.000
ln(Total text frequency of ego)	-50.311	3.273	-56.741	-43.968	17331	1.000
ln(Internet usage frequency of ego)	-0.763	2.954	-6.502	4.934	13027	1.000
ln(Phone retail price in Yuan)	11.132	2.909	5.563	16.875	11996	1.000
ln(Total WeChat frequency)	-14.786	3.608	-22.030	-7.853	15534	1.000
ln(Total frequency of other instant messaging)	-8.613	3.158	-14.629	-2.315	13200	1.000
Roaming dummy (1 = traveling outside of prefecture)	20.451	4.884	11.018	29.926	20108	1.000
Rural dummy (1 = rural)	21.360	4.754	11.993	30.774	20035	1.000
Damage dummy (1 = cell towers damaged in neighbourhood)	-1.783	5.016	-11.561	8.092	18686	1.000
Prior intra-family outbound call share	18.902	4.980	9.140	28.746	16404	1.000
(Intercept)	5.482	4.885	-4.106	15.113	17466	1.000

Notes. Number of obs. = 18,812; Controlled for random effects of families; Bayesian estimations produce credible intervals (CI), which are the intervals within which the parameter values fall at some particular probability.

Below, we provide the model diagnostics for the Bayesian Estimation of Model 2.

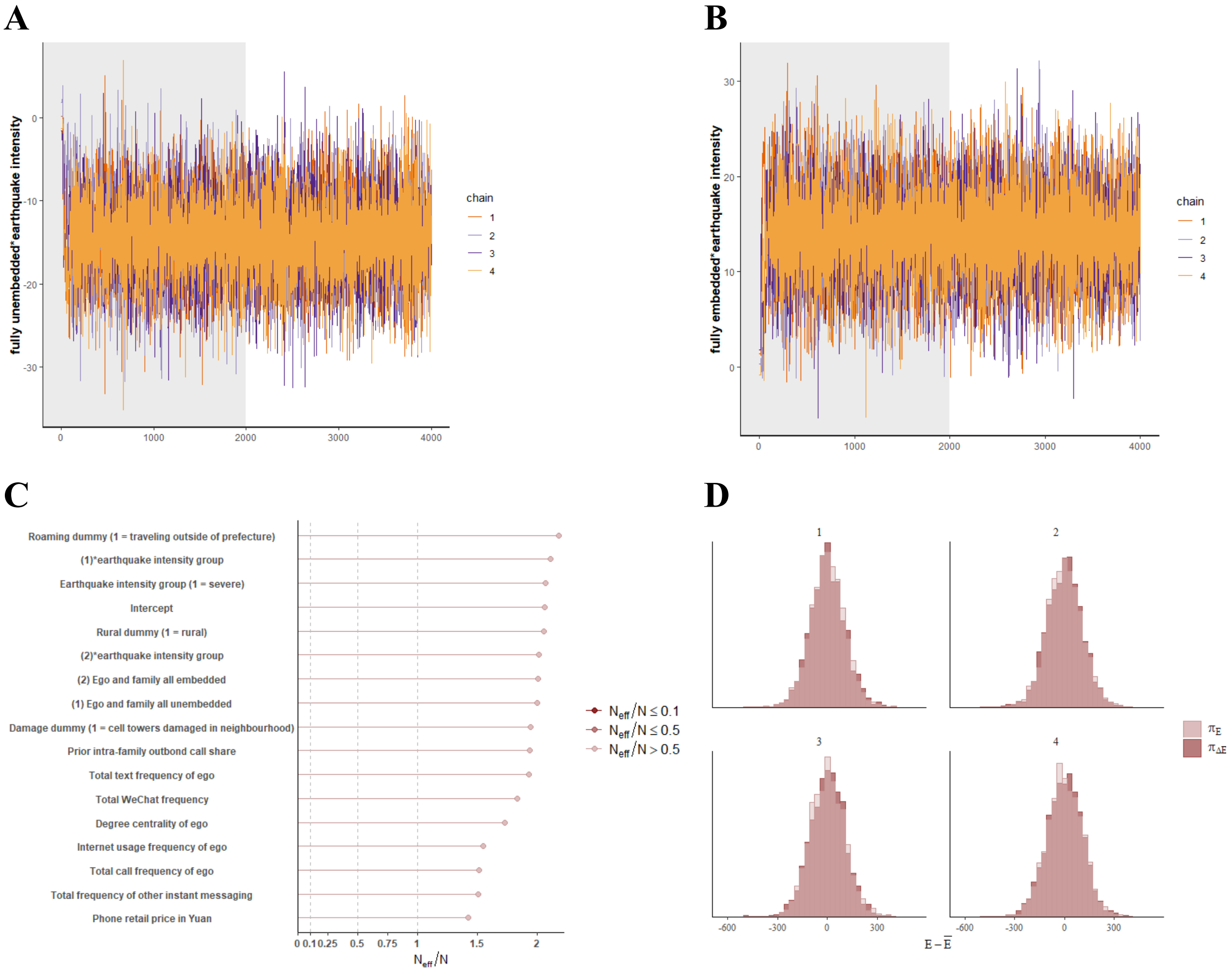

Supplementary Figure 10. Model diagnostics for Bayesian Estimation of Model 2. A and **B** are trace plots for the parameters of the two interaction terms; (**C**) The ratio of the effective sample sizes over the total sample sizes of parameters; (**D**) Overlaid histograms of the (centered) marginal energy distribution π_E and the first-differenced distribution $\pi_{\Delta E}$.

Roaming only robustness checks

Here we conduct the same analysis as Model 1, but restrict the sample only to users who were using roaming and thus outside of Ya'an (i.e., not home) at the time of the earthquake. We obtain analogous results, namely a positive interaction between fully unembedded family structures and earthquake intensity on likelihood of receiving a call from a non-family member first in the immediate aftermath of the earthquake.

Supplementary Table 7. Predicting probability that first outbound call is to a non-family tie (Roaming users only)

DV = *p*(first outbound call is to non-family plan member)	Coef.	S.E.	*z*	*p*>|*z*|	
(1) Ego and family all unembedded	0.485	0.171	2.83	0.005	**
(2) Ego and family all embedded	-0.323	0.069	-4.72	<0.001	***
Earthquake intensity group (1 = severe)	-0.100	0.085	-1.18	0.239	
(1)*earthquake intensity group	0.533	0.257	2.08	0.038	*
(2)*earthquake intensity group	0.107	0.104	1.03	0.303	
ln(Degree centrality of ego)	0.285	0.061	4.70	<0.001	***
ln(Total call frequency of ego)	-0.040	0.044	-0.91	0.365	
ln(Total text frequency of ego)	0.009	0.016	0.57	0.567	
ln(Internet usage frequency of ego)	-0.011	0.014	-0.75	0.456	
ln(Phone retail price in Yuan)	-0.011	0.034	-0.34	0.736	
ln(Total WeChat frequency)	-0.011	0.018	-0.64	0.520	
ln(Total frequency of other instant messaging)	-0.021	0.015	-1.39	0.164	
Rural dummy (1 = rural)	-0.202	0.054	-3.76	<0.001	***
Damage dummy (1 = cell towers damaged in neighbourhood)	0.240	0.165	1.46	0.146	
Prior intra-family outbound call share	-1.656	0.221	-7.49	<0.001	***
(Intercept)	0.173	0.233	0.74	0.458	

Notes. Number of obs. = 3,515; Controlled for random effects of families; The *p*-values are obtained from a two-sided *z*-test; $^{***}p < .001$, $^{**}p < .01$, $^{*}p < .05$, $^{\bullet}p < .1$

Panel models with earthquake shock

We combine panel data with exogenous shock of the earthquake, to examine how family embeddedness structure and earthquake jointly affect an ego's social network dynamics (Tables 3-4 in Main Text). Although individuals were not assigned to embeddedness structure groups, our model benefits from the shock of the earthquake which is exogenous. We make use of this fact to explore how different family embeddedness structure types respond to an earthquake shock.

Supplementary Table 8. Transition matrix of the three triads before and after the earthquake

Before \ After	Fully embedded triad	Non-fully embedded triad	Fully unembedded triad	Total
Fully embedded triad	62%	34%	4%	100%
Non-fully embedded triad	15%	66%	19%	100%
Fully unembedded triad	2%	28%	70%	100%

Family embeddedness structures are relatively stable (around 60% to 70% remained unchanged after quake). In particular, it is relatively rare for families where the ego and family are all unembedded to become fully embedded after the earthquake, and vice versa. This shows that the earthquake did not change the embeddedness structure for most families.

To examine how family embeddedness structure influences an ego's network during an earthquake, we directly compared individuals with fully embeddedness family structure (proposed experiment group) with those with fully unembedded family structure (proposed control group). Non-fully embedded triad is a transient state between fully embedded triad and fully unembedded triad. As Supplementary Table 8 shows, the choice of fully embedded triad (fully unembedded triad) as the experiment (control) avoids the switching of individuals from experiment (control) group to control (experiment) group after earthquake, therefore reducing the potential for confounding variables that drive both family embeddedness structure and the dependent variables.

The overall span of our data is from March 1st 2013 to June 30th 2013. Our dependent variables cover this entire period; however, due to data limitations, we can only observe five 1-week periods for the dependent variables. Putting these data together, we are able to construct a panel dataset with two periods of family embeddedness structure measurements before the earthquake (April 20th) and three periods of them after the earthquake. Each adjacent period covers four consecutive weeks before or after the earthquake. Specifically, period 1 covers 4-7 weeks before the earthquake (Mar. 2nd 8:02 am - Mar. 30th 8:01 am), period 2 covers 1-4 weeks before the earthquake (Mar. 23th 8:02 am - Apr. 20th 8:01 am), period 3 covers 1-4 weeks after

the earthquake (Apr. 20th 8:02 am - May.18th 8:01 am), period 4 covers 4-7 weeks after the earthquake (May.11th 8:02 am - Jun.8th 8:01 am), period 5 covers 7-10 weeks after the earthquake (Jun.1st 8:02 am - Jun.29th 8:01 am). There was slight overlap (1 week) between periods before the earthquake (period 1 and period 2) and periods after the earthquake (period 3 and period 4, period 4 and period 5) due to data limitations.

Supplementary Figure 11 suggests relatively parallel trends in the network outcome variables between the two groups before the earthquake (period 1 and period 2). While the occurrence of the earthquake increases the network outcome variables in both groups, the increase in magnitude is noticeably larger for the fully embedded group.

A **B**

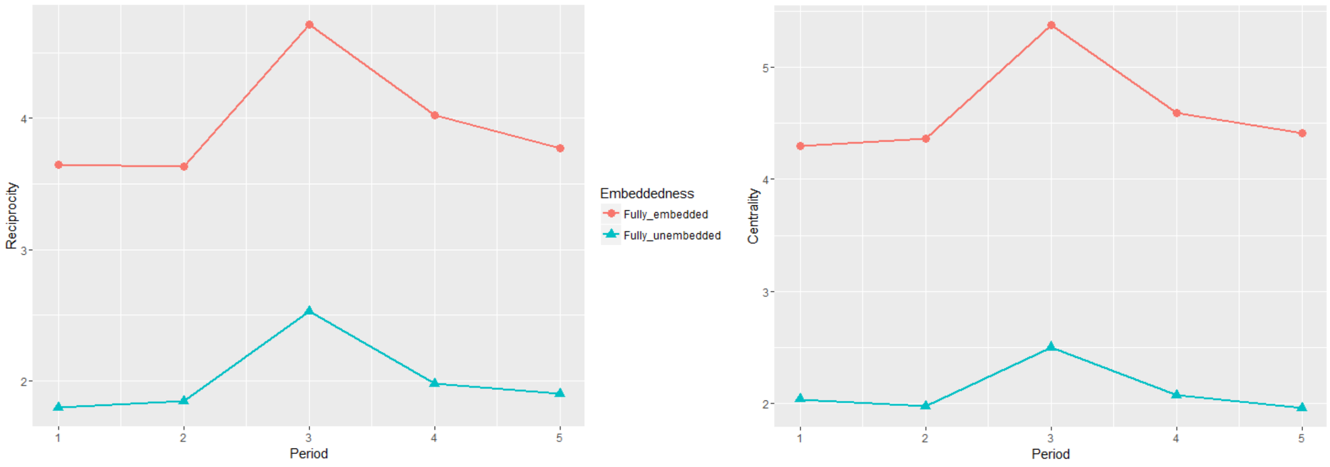

Supplementary Figure 11. Average of network outcome variables in experiment and control groups across time. Supplementary Figure 11 depicts the average of our network outcome variables of interest: reciprocity (**A**) and degree centrality (**B**), with respect to the two constructed family embeddedness triads across all five periods. Reciprocity is calculated as the total number of reciprocal calls between an ego and his two alters. The network outcome variables are measured respectively in the 4th week and 1st week before the earthquake, and 1st week, 4th week, and 7th week after the earthquake, in a symmetric manner with respect to the timing of earthquake.

To test the differential effects of the earthquake on ego's social network between the two family embeddedness triads, we set up panel data fixed effects model with the following equation:

$$Y_{ijt} = \beta_1 \text{Embedded}_{ijt} + \beta_2 \text{Earthquake}_t + \beta_3 (\text{Embedded}_{ijt} \times \text{Earthquake}_t) + \mathbf{C}_{ijt} \boldsymbol{\alpha}_1 + u_j + \epsilon_{ijt} \quad (1)$$

Where Y_{ijt} is the network outcome variable (reciprocity or degree centrality) for individual i in family j that is at period t. Embedded_{ijt} is a dummy variable that takes value one if individual i belongs to experiment group (i.e. fully embedded triad) at period t. Earthquake_t is a dummy variable indicating whether the period is after the earthquake (i.e. equals one after period 2). $\mathbf{C}_{ijt}$ is a vector of controlling variables including ego's degree centrality (for reciprocity outcome), ego's call, text, and Internet frequency, phone price, whether ego used instant messaging, whether ego was out of town during earthquake (roaming), whether ego lives in a rural district, and whether the telecom tower was damaged during earthquake. u_j are family fixed effects, controlling for potentially unobserved family-level factors. ϵ_{ijt} is a random, idiosyncratic error term. β_3 is our coefficient of interest, examining the differential effects of fully embedded triads and fully unembedded triads (on network outcome) during earthquake.

β_3 is significantly positive for both network outcomes, suggesting that higher earthquake intensity results in greater reciprocity and higher centrality for individuals with fully embedded family structure, compared to those with fully unembedded family structure.

Other independent variables. More active communicators were also more reciprocal with their families. People with more friends had fewer interactions between family members, which might reflect communications capacity constraints. The positive sign of phone retail price is difficult to interpret since reflects both higher social economic status and greater phone (communications) functionality. The positive sign for WeChat usage suggests that the most popular messaging app in China strengthened rather than weakened family communications. Likewise, the negative signs for being physically away (roaming) or residing in rural regions suggests that they were impediments to reciprocal communications.

Individual-Fixed Effects Robustness checks.

To further control for the effects of unmeasured individual-specific and time-specific characteristics, we re-estimated the model by further including individual fixed effects and period fixed effects. The estimation results are consistent with the base model.

Supplementary Table 9. Predicting reciprocity (Model 3) with individual fixed effects

DV = Reciprocity between ego and two alters	Coef.	Cluster S.E.	*t*	*p*>|*t*|	
Ego and family all embedded	NA	NA	NA	NA	
Earthquake dummy (1 = post-quake)	NA	NA	NA	NA	
Ego and family all embedded * Earthquake dummy	0.152	0.049	3.13	0.002	**
ln(Degree centrality of ego)	-0.835	0.052	-15.99	<0.001	***
ln(Total call frequency of ego)	1.305	0.029	45.02	<0.001	***
ln(Total text frequency of ego)	0.159	0.020	7.79	<0.001	***
ln(Internet usage frequency of ego)	0.004	0.006	0.66	0.508	
ln(Phone retail price in Yuan)	NA	NA	NA	NA	
ln(Total WeChat frequency)	NA	NA	NA	NA	
ln(Total frequency of other instant messaging)	NA	NA	NA	NA	
Roaming dummy (1 = traveling outside of prefecture)	NA	NA	NA	NA	
Rural dummy (1 = rural)	NA	NA	NA	NA	
Damage dummy (1 = cell towers damaged in neighbourhood)	NA	NA	NA	NA	
Prior intra-family outbound call share	NA	NA	NA	NA	
Individual fixed effects	Yes				

Notes. Number of obs. = 49,851; Time periods = 5; The *p*-values are obtained from a two-sided *t*-test; $^{***}p < .001$, $^{**}p < .01$, $^{*}p < .05$, $^{\bullet}p < .1$

(1) Controlled for fixed effects of individuals and periods.

(2) Individual-invariant variables and time-invariant variables are perfectly collinear with individual and period fixed effects respectively, and thus were excluded from model estimation

(3) Hausman test results preferred fixed effects model (χ^2 = 315.02, df = 5, $p < .001$)

Supplementary Table 10. Predicting degree centrality (Model 4) with individual fixed effects

DV = Ego's centrality	Coef.	Cluster S.E.	*t*	*p*>|*t*|	
Ego and family all embedded	NA	NA	NA	NA	
Earthquake dummy (1 = post-quake)	NA	NA	NA	NA	
Ego and family all embedded * Earthquake dummy	0.257	0.037	6.86	<0.001	***
ln(Total call frequency of ego)	0.830	0.017	47.74	<0.001	***
ln(Total text frequency of ego)	0.175	0.016	11.09	<0.001	***
ln(Internet usage frequency of ego)	-0.007	0.005	-1.37	0.171	
ln(Phone retail price in Yuan)	NA	NA	NA	NA	
ln(Total WeChat frequency)	NA	NA	NA	NA	
ln(Total frequency of other instant messaging)	NA	NA	NA	NA	
Roaming dummy (1 = traveling outside of prefecture)	NA	NA	NA	NA	
Rural dummy (1 = rural)	NA	NA	NA	NA	
Damage dummy (1 = cell towers damaged in neighbourhood)	NA	NA	NA	NA	
Prior intra-family outbound call share	NA	NA	NA	NA	
Individual fixed effects	Yes				

Notes. Number of obs. = 49,851; Time periods = 5; The *p*-values are obtained from a two-sided *t*-test; $^{***}p < .001$, $^{**}p < .01$, $^{*}p < .05$, $^{\bullet}p < .1$

(1) Controlled for fixed effects of individuals and periods.

(2) Individual-invariant variables and time-invariant variables are perfectly collinear with individual and period fixed effects respectively, and thus were excluded from model estimation

(3) Hausman test results preferred fixed effects model (χ^2 = 751.50, df = 4, $p < .001$)

Embeddedness strength manipulation checks

In the Supplementary Tables 11-14, mean of family embeddedness is defined as mean number of common friends (overlap) of the entire family. The purpose of this robustness check is to show that the analyses in the main text, which uses a dummy variable for family structure, persists when we use a continuous measurement (overlap parameter of the entire 3-person family) to operationalize family embeddedness. This alleviates concerns that our main results were a statistical artefact of the dichotomous operationalization of embeddedness.

Supplementary Table 11: Predicting initial outbound calls with embeddedness strength

DV = *p*(first outbound call is to non-family plan member)	Coef.	S.E.	*z*	*p*>|*z*|	
Mean of family embeddedness	-0.050	0.006	-8.50	<0.001	***
Earthquake intensity group (1 = severe)	0.148	0.033	4.46	<0.001	***
Mean of family embeddedness * earthquake intensity group	-0.016	0.009	-1.68	0.093	•
ln(Degree centrality of ego)	0.179	0.024	7.43	<0.001	***
ln(Total call frequency of ego)	-0.069	0.018	-3.78	<0.001	***
ln(Total text frequency of ego)	0.009	0.007	1.38	0.168	
ln(Internet usage frequency of ego)	-0.006	0.006	-1.08	0.280	
ln(Phone retail price in Yuan)	-0.015	0.013	-1.10	0.271	
ln(Total WeChat frequency)	0.002	0.007	0.28	0.778	
ln(Total frequency of other instant messaging)	0.001	0.006	0.09	0.931	
Roaming dummy (1 = traveling outside of prefecture)	-0.365	0.027	-13.59	<0.001	***
Rural dummy (1 = rural)	-0.093	0.022	-4.31	<0.001	***
Damage dummy (1 = cell towers damaged in neighbourhood)	0.311	0.072	4.35	<0.001	***
Prior intra-family outbound call share	-2.013	0.074	-27.34	<0.001	***
(Intercept)	0.886	0.092	9.62	<0.001	***

Notes. Number of obs. = 26,394; Controlled for random effects of families; The *p*-values are obtained from a two-sided *z*-test; $^{***}p < .001$, $^{**}p < .01$, $^{*}p < .05$, $^{\bullet}p < .1$

Consistent with Table 1, higher level(s) of embeddedness has a negative interaction effect with earthquake intensity on probability that the first outbound call is to a non-family plan member.

Supplementary Table 12: Predicting first outbound call latency with embeddedness strength

DV = Hours until first outbound call to non-family plan member	Coef.	S.D.	Lower 2.5% CI	Upper 97.5% CI	N_eff	Rhat
Mean of family embeddedness	33.601	3.856	25.921	41.033	15108	1.000
Earthquake intensity group (1 = severe)	-15.311	4.603	-24.188	-6.297	17522	1.000
Mean of common friends * earthquake intensity group	12.726	4.099	4.665	20.659	14745	1.000
ln(Degree centrality of ego)	-58.146	4.293	-66.719	-49.722	15318	1.000
ln(Total call frequency of ego)	-34.698	3.748	-41.993	-27.425	12119	1.000
ln(Total text frequency of ego)	-22.233	3.320	-28.807	-15.791	15477	1.000
ln(Internet usage frequency of ego)	2.750	3.004	-3.150	8.610	14515	1.000
ln(Phone retail price in Yuan)	-0.451	2.910	-6.102	5.103	12684	1.000
ln(Total WeChat frequency)	-3.979	3.466	-10.775	2.757	15380	1.000
ln(Total frequency of other instant messaging)	-11.805	3.104	-17.867	-5.819	13260	1.000
Roaming dummy (1 = traveling outside of prefecture)	19.668	4.817	10.359	29.235	18709	1.000
Rural dummy (1 = rural)	14.903	4.692	5.914	24.048	19286	1.000
Damage dummy (1 = cell towers damaged in neighbourhood)	-1.744	4.949	-11.297	7.639	18135	1.000
Prior intra-family outbound call share	24.641	4.920	15.012	34.469	17354	1.000
(Intercept)	2.056	4.945	-7.415	11.729	18751	1.000

Notes. Number of obs. = 18,958; Controlled for random effects of families; Bayesian estimations produce credible intervals (CI), which are the intervals within which the parameter values fall at some particular probability.

Here, consistent with Table 2, higher level(s) of embeddedness has a positive interaction effect with earthquake intensity on temporal latency of the first outbound call to a non-family plan member.

Supplementary Table 13: Predicting reciprocity with embeddedness strength

DV = Reciprocity	Coef.	Cluster S.E.	*t*	*p*>|*t*|	
Mean of family embeddedness	0.370	0.071	5.20	<0.001	***
Earthquake dummy (1 = post-quake)	0.206	0.036	5.70	<0.001	***
Mean of family embeddedness * Earthquake dummy	0.064	0.031	2.05	0.041	*
ln(Degree centrality of ego)	-0.792	0.058	-13.75	<0.001	***
ln(Total call frequency of ego)	1.320	0.039	33.74	<0.001	***
ln(Total text frequency of ego)	0.114	0.027	4.17	<0.001	***
ln(Internet usage frequency of ego)	0.016	0.006	2.58	0.010	**
ln(Phone retail price in Yuan)	0.179	0.040	4.51	<0.001	***
ln(Total WeChat frequency)	0.010	0.021	0.47	0.638	
ln(Total frequency of other instant messaging)	-0.020	0.016	-1.30	0.195	
Roaming dummy (1 = traveling outside of prefecture)	-0.059	0.079	-0.74	0.457	
Rural dummy (1 = rural)	0.110	0.184	0.60	0.551	
Damage dummy (1 = cell towers damaged in neighbourhood)	-0.741	0.541	-1.37	0.170	
Prior intra-family outbound call share	-0.104	0.193	-0.54	0.591	
Family fixed effects	Yes				

Notes. Number of obs. = 49,851; Controlled for fixed effects of families; Time periods = 5; The *p*-values are obtained from a two-sided *t*-test; $^{***}p < .001$, $^{**}p < .01$, $^{*}p < .05$, $^{\bullet}p < .1$; The standard errors were clustered at the family levels

Here, consistent with Table 3, higher level(s) of embeddedness has a positive interaction effect with earthquake intensity on reciprocity.

Supplementary Table 14: Predicting degree centrality with embeddedness strength

DV = Ego's centrality	Coef.	Cluster S.E.	*t*	*p*>|*t*|	
Mean of family embeddedness	0.355	0.092	3.87	<0.001	***
Earthquake dummy (1 = post-quake)	0.027	0.035	0.77	0.439	
Mean of family embeddedness * Earthquake dummy	0.144	0.047	3.04	0.002	**
ln(Total call frequency of ego)	1.547	0.038	40.79	<0.001	***
ln(Total text frequency of ego)	0.381	0.042	9.08	<0.001	***
ln(Internet usage frequency of ego)	-0.008	0.008	-1.10	0.273	
ln(Phone retail price in Yuan)	0.349	0.050	6.94	<0.001	***
ln(Total WeChat frequency)	0.100	0.034	2.90	0.004	***
ln(Total frequency of other instant messaging)	-0.030	0.021	-1.39	0.163	
Roaming dummy (1 = traveling outside of prefecture)	-0.653	0.088	-7.41	<0.001	***
Rural dummy (1 = rural)	-0.052	0.204	-0.26	0.799	
Damage dummy (1 = cell towers damaged in neighbourhood)	-0.091	0.825	-0.11	0.912	
Prior intra-family outbound call share	-4.361	0.234	-18.67	<0.001	***
Family fixed effects	Yes				

Notes. Number of obs. = 49,851; Controlled for fixed effects of families; Time periods = 5; The *p*-values are obtained from a two-sided *t*-test; $^{***}p < .001$, $^{**}p < .01$, $^{*}p < .05$, $^{\bullet}p < .1$; The standard errors were clustered at the family levels

Here, consistent with Table 4, higher level(s) of embeddedness has a positive interaction effect with earthquake intensity on degree centrality.

Variance of Family Embeddedness

Finally, we investigate how variance of family embeddedness affects social dynamics (Supplementary Fig. 7, Supplementary Tables 15-18) using analogous statistical models (same dependent variables as Models 1-4). These analyses showcase how additional insights can be gleaned from a structural and triadic conceptualization of embeddedness that are otherwise unobservable from a purely dyadic perspective. In addition, we demonstrate that the measure generates additional insights on embeddedness structure that the dummy variables in our previous analyses cannot provide.

We define variance of family embeddedness as the variance of the number of embedded non-family friends that each *dyad* (i.e., edge or link between two nodes) in the family triad has. This measure reflects the relative imbalance in shared social ties in a family; e.g., in a balanced structure where all 3 family dyads have similar numbers of embedded ties, variance is low; in an unbalanced structure where one or two dyads have many more embedded ties, variance is high. The variance measure has the advantage of reflecting the weighting or relative differences in the strength of embeddedness of the different family dyads. It is noteworthy how this measure differs from a family's 'embeddedness strength', which may be operationalized as the *mean* number of embedded non-family ties in each dyad (and does not require a triadic perspective). For example, if Family A has 3 dyads that all have 2 embedded ties, and Family B has two dyads with 1 embedded tie, and one dyad with 4 embedded ties, then Family A and B have the same mean family embeddedness (2), but different variance in family embeddedness (6).

We first use the same statistical approach as Models 1-2 to test the interaction between variance of family embeddedness and the embeddedness structure dummy. We find that egos in fully embedded family structures are even less (more) likely to call friends (family) rather than family first (Supplementary Table 15). We also find that egos in fully embedded family structures are relatively slower in activating their non-family social network if their family has higher variance in embeddedness (Supplementary Table 16). Both results suggest that egos in fully embedded family structures are even more likely to prioritize family communications where there is imbalance in embeddedness within the family. One possible explanation for these results is that in fully embedded family structures, imbalance signals the presence of one particularly close dyadic relationship, which received relative prioritization immediately after the earthquake.

We then use the same statistical approach as Models 3-4 and explore how the mean and variance of family embeddedness affect post-earthquake social dynamics. We find a significant negative interaction effect between mean and variance of family embeddedness for both reciprocity and centrality (Supplementary Tables 17-18). In other words, a family triad with more shared social resources will still have *less* reciprocity and lower degree centrality if it is unbalanced in embeddedness structure. This qualification of the benefit of greater embeddedness strength (i.e., overlap parameter) again underscores the relative advantages of a triadic, structural perspective (and operationalization) of structural embeddedness over a purely dyadic (and frequency-based) measurement.

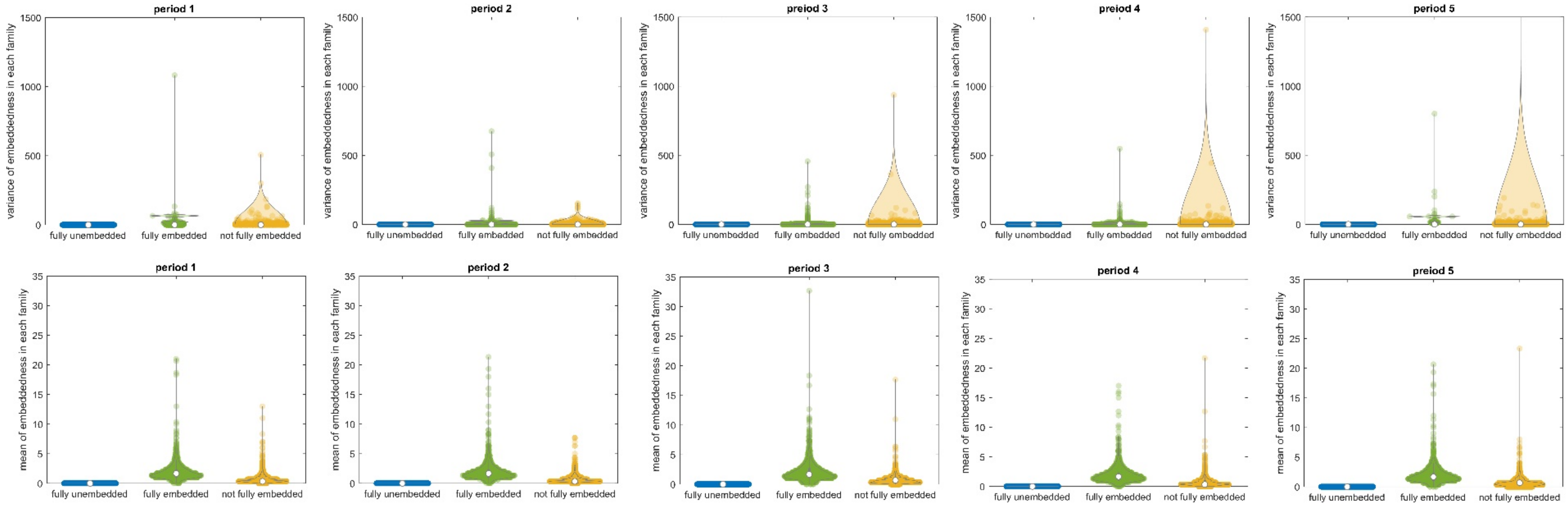

Supplementary Figure 12. Stability of mean and variance of embeddedness structure.

Note: Earthquake occurred between period 2 and period 3.

Supplementary Table 15: Predicting initial outbound calls with variance of family embeddedness

DV = *p*(1st outbound call is to non-family plan member)	Coef.	SE	*z*	*p*>|*z*|	
(1) Ego and family all unembedded	0.677	0.081	8.33	<0.001	***
(2) Ego and family all embedded	-0.163	0.040	-4.09	<0.001	***
Variance of Family Embeddedness	0.009	0.004	2.41	0.016	
(1)*Variance of Family Embeddedness	NA	NA	NA	NA	
(2)*Variance of Family Embeddedness	-0.011	0.004	-2.60	0.009	**
Earthquake intensity group (1 = severe)	0.046	0.029	1.58	0.114	
ln(Degree centrality of ego)	0.181	0.036	4.99	<0.001	***
ln(Total call frequency of ego)	-0.096	0.027	-3.56	<0.001	***
ln(Total text frequency of ego)	0.005	0.010	0.48	0.630	
ln(Internet usage frequency of ego)	-0.008	0.009	-0.93	0.350	
ln(Phone retail price in Yuan)	-0.033	0.020	-1.63	0.103	
ln(Total WeChat frequency)	0.001	0.011	0.10	0.924	
ln(Total frequency of other instant messaging)	0.011	0.010	1.20	0.232	
Roaming dummy (1 = outside of Ya'an)	-0.364	0.040	-9.12	<0.001	***
Rural dummy (1 = rural)	-0.094	0.031	-3.07	0.002	**
Damage dummy (1 = cell towers damaged)	0.327	0.105	3.13	0.002	**
Prior intra-family outbound call share	-1.746	0.110	-15.86	<0.001	***
(Intercept)	0.981	0.139	7.08	<0.001	***

Notes. Number of obs. = 26,394; The *p*-values are obtained from a two-sided *z*-test; $^{***}p < .001$, $^{**}p < .01$, $^{*}p < .05$, $^{\bullet}p < .1$; Controlled for random effects of families

Supplementary Table 16: Predicting first outbound call latency with variance of family embeddedness

DV = Hours until first outbound call to non-family plan member	Coef.	S.D.	Lower 2.5% CI	Upper 97.5% CI	N_eff	Rhat
(1) Ego and family all unembedded	-26.868	4.8742	-36.294	-17.407	21319	1.000
(2) Ego and family all embedded	56.376	4.6833	46.977	65.503	18289	1.000
Variance of Family Embeddedness	4.665	1.9007	0.831	8.409	16979	1.000
(1)*Variance of Family Embeddedness	NA	NA	NA	NA	NA	NA
(2)*Variance of Family Embeddedness	4.713	1.8972	0.951	8.449	19865	1.000
Earthquake intensity group (1 = severe)	-15.671	4.6131	-24.648	-6.729	16666	1.000
ln(Degree centrality of ego)	-56.854	4.2233	-65.147	-48.660	15506	1.000
ln(Total call frequency of ego)	-34.710	3.7952	-42.140	-27.229	14230	1.000
ln(Total text frequency of ego)	-20.993	3.234	-27.276	-14.544	16492	1.000
ln(Internet usage frequency of ego)	2.938	2.9127	-2.856	8.730	13988	1.000
ln(Phone retail price in Yuan)	-4.621	2.8348	-10.137	0.849	13127	1.000
ln(Total WeChat frequency)	-3.499	3.4981	-10.160	3.440	16958	1.000
ln(Total frequency of other instant messaging)	-11.806	3.0842	-17.906	-5.763	12708	1.000
Roaming dummy (1 = outside of Ya'an)	19.610	4.9585	9.940	29.184	20666	1.000
Rural dummy (1 = rural)	14.567	4.6512	5.320	23.740	18466	1.000
Damage dummy (1 = cell towers damaged)	-1.778	5.0173	-11.280	8.044	17044	1.000
Prior intra-family outbound call share	24.639	4.8741	15.168	33.928	18817	1.000
(Intercept)	1.218	4.913	-8.456	10.971	19747	1.000

Notes. Number of obs. = 18,958; Controlled for random effects of families; Bayesian estimations produce credible intervals (CI), which are the intervals within which the parameter values fall at some particular probability; The values of the interaction between "Ego and family all unembedded" and "Variance of common friends" are all zero (By definition, variance of family embeddedness when ego and family are all unembedded is always zero), and thus was excluded from model estimation

Supplementary Table 17: Predicting reciprocity with variance of family embeddedness

DV = Reciprocity	Coef.	Cluster S.E.	*t*	*p*>|*t*|	
Mean of Family Embeddedness	0.373	0.075	5.00	<0.001	***
Variance of Family Embeddedness	0.016	0.009	1.78	0.075	·
Mean of common friends * Variance of common friends	-0.001	<0.001	-2.70	0.007	**
Earthquake dummy (1 = post-quake)	0.246	0.036	6.78	<0.001	***
ln(Degree centrality of ego)	-0.732	0.056	-13.08	<0.001	***
ln(Total call frequency of ego)	1.282	0.038	33.86	<0.001	***
ln(Total text frequency of ego)	0.123	0.027	4.55	<0.001	***
ln(Internet usage frequency of ego)	0.015	0.006	2.37	0.018	*
ln(Phone retail price in Yuan)	0.175	0.039	4.54	<0.001	***
ln(Total WeChat frequency)	0.009	0.020	0.46	0.643	
ln(Total frequency of other instant messaging)	-0.022	0.016	-1.41	0.158	
Roaming dummy (1 = traveling outside of Ya'an)	-0.045	0.074	-0.61	0.543	
Rural dummy (1 = rural)	0.144	0.175	0.82	0.411	
Damage dummy (1 = cell towers damaged)	-1.253	0.350	-3.58	<0.001	***
Prior intra-family outbound call share	-0.087	0.186	-0.47	0.641	
Family fixed effects	Yes				

Notes. Number of obs. = 49,851; Time periods = 5; The *p*-values are obtained from a two-sided *t*-test; $^{***}p < .001$, $^{**}p < .01$, $^{*}p < .05$, $^{\bullet}p < .1$; Controlled for fixed effects of families; The standard errors were clustered at the family levels.

Supplementary Table 18: Predicting degree centrality with variance of family embeddedness

DV = Ego's centrality	Coef.	Cluster S.E.	*t*	*p*>|*t*|	
Mean of Family Embeddedness	0.277	0.057	4.86	<0.001	***
Variance of Family Embeddedness	0.065	0.014	4.66	<0.001	***
Mean of common friends * Variance of common friends	-0.002	0.001	-4.09	<0.001	***
Earthquake dummy (1 = post-quake)	0.145	0.027	5.41	<0.001	***
ln(Total call frequency of ego)	1.552	0.037	42.13	<0.001	***
ln(Total text frequency of ego)	0.414	0.044	9.50	<0.001	***
ln(Internet usage frequency of ego)	-0.008	0.008	-0.95	0.344	
ln(Phone retail price in Yuan)	0.341	0.054	6.28	<0.001	***
ln(Total WeChat frequency)	0.110	0.036	3.09	0.002	**
ln(Total frequency of other instant messaging)	-0.040	0.023	-1.76	0.079	
Roaming dummy (1 = traveling outside of Ya'an)	-0.640	0.087	-7.35	<0.001	***
Rural dummy (1 = rural)	0.013	0.174	0.08	0.940	
Damage dummy (1 = cell towers damaged)	-0.110	0.566	-0.19	0.847	
Prior intra-family outbound call share	-4.298	0.223	-19.31	<0.001	***
Family fixed effects	Yes				

Notes. Number of obs. = 49,851; Time periods = 5; The *p*-values are obtained from a two-sided *t*-test; $^{***}p < .001$, $^{**}p < .01$, $^{*}p < .05$, $^{\bullet}p < .1$; Controlled for fixed effects of families; The standard errors were clustered at the family levels.

Miscellaneous Robustness Checks

4-person families. In Supplementary Tables 19-20 below, we test Models 1 and 2 for 4 person families. Our primary results focused on family triads for conceptual and empirical reasons. Nonetheless, one might wonder if larger family sizes had different social dynamics in the aftermath of the earthquake. Here, we collected additional data for families with 4 members (2223 families in total) and re-estimated Models 1 and 2.

The results were both consistent with that of the baseline models in both signs and significance, suggesting that our findings are applicable beyond families of three members; i.e., an ego whose family is more embedded (regardless if the family has 3 or 4 members) is generally less likely to call non-family members after an earthquake, and it also takes longer time for him/her to do so.

Supplementary Table 19: Robustness check for Model 1 using 4 person families

Dependent Variable = *p*(first outbound call is to non-family plan member)	Coef.	S.E.	*z*	*p*>|*z*|	
(1) Ego and family all unembedded	0.306	0.135	2.26	0.024	*
(2) Ego and family all embedded	-0.051	0.052	-0.97	0.330	
Earthquake intensity group (1 = severe)	0.069	0.056	1.24	0.214	
(1)*earthquake intensity group	1.293	0.445	2.90	0.004	**
(2)*earthquake intensity group	-0.082	0.123	-0.67	0.503	
ln(Degree centrality of ego)	0.020	0.044	0.45	0.655	
ln(Total call frequency of ego)	0.194	0.031	6.29	<0.001	***
ln(Total text frequency of ego)	-0.046	0.013	-3.58	<0.001	***
ln(Internet usage frequency of ego)	-0.016	0.012	-1.30	0.194	
ln(Phone retail price in Yuan)	0.008	0.023	0.35	0.725	
ln(Total WeChat frequency)	0.058	0.068	0.85	0.395	
ln(Total frequency of other instant messaging)	-0.058	0.122	-0.48	0.632	
Roaming dummy (1 = traveling outside of prefecture)	-0.241	0.069	-3.49	<0.001	***
Rural dummy (1 = rural)	-0.003	0.039	-0.07	0.948	
Damage dummy (1 = cell towers damaged in neighborhood)	0.103	0.091	1.14	0.256	
Prior intra-family outbound call share	-1.531	0.077	-19.93	<0.001	***
(Intercept)	-0.054	0.154	-0.35	0.724	

Notes. Number of obs. = 7,515; Controlled for random effects of families; The *p*-values are obtained from a two-sided *z*-test; $^{***}p < .001$, $^{**}p < .01$, $^{*}p < .05$, $^{\bullet}p < .1$

Supplementary Table 20: Robustness check for Model 2 using 4 person families

Dependent Variable = Time until first outbound call to non-family plan member (hours)	Coef.	S.D.	Lower 2.5% CI	Upper 97.5% CI	N_eff	Rhat
(1) Ego and family all unembedded	-10.710	4.954	-20.287	-1.032	23764	1.000
(2) Ego and family all embedded	12.579	4.660	3.504	21.741	18355	1.000
Earthquake intensity group (1 = severe)	-7.998	3.765	-15.321	-0.663	18602	1.000
(1)*earthquake intensity group	-5.463	2.003	-9.324	-1.562	19241	1.000
(2)*earthquake intensity group	5.272	1.990	1.277	9.143	19233	1.000
ln(Degree centrality of ego)	-29.732	4.232	-38.074	-21.392	12984	1.000
ln(Total call frequency of ego)	-20.462	3.326	-26.948	-13.955	13356	1.000
ln(Total text frequency of ego)	-9.273	2.879	-14.966	-3.656	17570	1.000
ln(Internet usage frequency of ego)	-4.719	2.473	-9.592	0.089	14082	1.000
ln(Phone retail price in Yuan)	17.314	2.242	12.934	21.738	12748	1.000
ln(Total WeChat frequency)	0.846	3.617	-6.289	7.945	11842	1.000
ln(Total frequency of other instant messaging)	0.823	4.656	-8.237	9.897	14312	1.000
Roaming dummy (1 = traveling outside of prefecture)	2.142	4.723	-7.181	11.384	17735	1.000
Rural dummy (1 = rural)	5.684	4.511	-2.984	14.367	18780	1.000
Damage dummy (1 = cell towers damaged in neighborhood)	-0.937	4.693	-10.166	8.152	18733	1.000
Prior intra-family outbound call share	33.235	4.899	23.521	42.769	18614	1.000
(Intercept)	6.697	4.971	-3.346	16.490	18334	1.000

Notes. Number of obs. = 4,118; Controlled for random effects of families. Bayesian estimations produce credible intervals (CI), which are the intervals within which the parameter values fall at some particular probability.

Type 1 vs. Type 2. One may wonder what the difference between a family where an ego is non-embedded with two family members who are embedded (Type 2), and a family where all members are unembedded (Type 1). Here, we directly compare the differential impact of these two family structures (with the fully unembedded group as the reference).

The results suggest that when ego is unembedded but alters are embedded, the ego was less likely to call non-family members after an earthquake (Supplementary Table 21), and also took longer to do so (Supplementary Table 22), as compared to when ego and alters were all unembedded. This shows that the two family structures are different in their social response to the earthquake.

The general rationale is that the relationship of the other family members may also influence the ego's behavior. For example, it is possible that parents who have a strong rather than weak relationship with each other (e.g., if the two family members are embedded or not) are more likely to influence their adult child.

Supplementary Table 21: Robustness check comparing family Type 1 vs. 2 for Model 1

Dependent Variable = *p*(first outbound call is to non-family plan member)	Coef.	S.E.	*z*	*p*>|*z*|	
(1) Ego unembedded alters embedded (Type 2)	-0.128	0.156	-0.83	0.409	
Earthquake intensity group (1 = severe)	0.269	0.098	2.75	0.006	**
(1)*earthquake intensity group	-0.445	0.220	-2.02	0.043	*
ln(Degree centrality of ego)	0.102	0.093	1.10	0.271	
ln(Total call frequency of ego)	0.093	0.064	1.45	0.146	
ln(Total text frequency of ego)	0.006	0.032	0.19	0.849	
ln(Internet usage frequency of ego)	-0.010	0.026	-0.38	0.706	
ln(Phone retail price in Yuan)	-0.029	0.060	-0.49	0.627	
ln(Total WeChat frequency)	0.020	0.033	0.60	0.548	
ln(Total frequency of other instant messaging)	-0.003	0.028	-0.12	0.904	
Roaming dummy (1 = traveling outside of prefecture)	-0.395	0.114	-3.45	<0.001	***
Rural dummy (1 = rural)	-0.212	0.096	-2.22	0.026	*
Damage dummy (1 = cell towers damaged in neighborhood)	0.910	0.458	1.99	0.047	*
Prior intra-family outbound call share	-2.672	0.289	-9.24	<0.001	***
(Intercept)	1.044	0.395	2.64	0.008	**

Notes. Number of obs. = 2,132; Controlled for random effects of families; The *p*-values are obtained from a two-sided *z*-test; $^{***}p < .001$, $^{**}p < .01$, $^{*}p < .05$, $^{\bullet}p < .1$

Supplementary Table 22: Robustness check comparing family Type 1 vs. 2 for Model 2

Dependent Variable = Time until first outbound call to non-family plan member (hours)	Coef.	S.D.	Lower 2.5% CI	Upper 97.5% CI	N_eff	Rhat
(1) Ego unembedded alters embedded (Type 2)	11.963	4.915	2.410	21.652	10733	1.000
Earthquake intensity group (1 = severe)	-6.298	2.990	-12.095	-0.376	12134	1.000
(1)*earthquake intensity group	12.214	4.957	2.393	21.772	11405	1.000
ln(Degree centrality of ego)	-36.844	4.605	-45.844	-27.766	9567	1.000
ln(Total call frequency of ego)	-47.743	4.095	-55.861	-39.640	7549	1.000
ln(Total text frequency of ego)	-20.514	4.162	-28.820	-12.625	9918	1.000
ln(Internet usage frequency of ego)	-7.555	3.735	-14.915	-0.295	9847	1.000
ln(Phone retail price in Yuan)	23.518	3.200	17.282	29.696	6797	1.001
ln(Total WeChat frequency)	-9.532	4.420	-18.222	-0.942	11374	1.000
ln(Total frequency of other instant messaging)	-14.806	3.994	-22.756	-7.188	10081	1.000
Roaming dummy (1 = traveling outside of prefecture)	4.506	5.005	-5.297	14.060	10821	1.000
Rural dummy (1 = rural)	9.441	4.928	0.004	19.058	11396	1.000
Damage dummy (1 = cell towers damaged in neighborhood)	-1.006	4.975	-10.728	8.698	11726	1.000
Prior intra-family outbound call share	7.440	5.001	-2.498	17.152	10365	1.000
(Intercept)	4.687	5.118	-5.232	14.841	11300	1.000

Notes. Number of obs. = 1,509; Controlled for random effects of families. Bayesian estimations produce credible intervals (CI), which are the intervals within which the parameter values fall at some particular probability.

Additional controls. In Supplementary Tables 23 and 25, we repeat the analyses of Tables 1 and 2 of the main text, respectively after controlling for dyadic embeddedness (overlap parameter) with the important tie and ln(tie strength with the important tie). We do not use these in the main text because they are highly correlated with the dependent variables.

Supplementary Table 23: Robustness Check for Table 1 including dyadic embeddedness and tie strength with important tie

Dependent Variable = *p(*first outbound call is to non-family plan member*)*	Coef.	S.E.	*z*	*p*>|*z*|	
(1) Ego and family all unembedded	0.548	0.071	7.76	<0.001	***
(2) Ego and family all embedded	-0.292	0.029	-10.00	<0.001	***
Earthquake intensity group (1 = severe)	0.089	0.037	2.38	0.017	*
(1)*earthquake intensity group	0.241	0.110	2.19	0.028	*
(2)*earthquake intensity group	-0.011	0.045	-0.24	0.814	
ln(Degree centrality of ego)	0.056	0.026	2.16	0.030	*
ln(Total call frequency of ego)	0.222	0.020	11.26	<0.001	***
ln(Total text frequency of ego)	0.012	0.007	1.67	0.095	.
ln(Internet usage frequency of ego)	-0.009	0.006	-1.34	0.179	
ln(Phone retail price in Yuan)	-0.005	0.014	-0.38	0.707	
ln(Total WeChat frequency)	0.007	0.008	0.88	0.381	
ln(Total frequency of other instant messaging)	0.003	0.007	0.51	0.611	
Roaming dummy (1 = traveling outside of prefecture)	-0.357	0.028	-12.58	<0.001	***
Rural dummy (1 = rural)	-0.081	0.022	-3.62	<0.001	***
Damage dummy (1 = cell towers damaged in neighborhood)	0.255	0.076	3.37	<0.001	***
Prior intra-family callout share	-1.534	0.078	-19.67	<0.001	***
Dyadic embeddedness with important tie	-0.371	0.011	-34.86	<0.001	***
Tie strength with important tie	-0.451	0.012	-36.88	<0.001	***
(Intercept)	-0.176	0.098	-1.79	0.074	.

Notes. Number of obs. = 26,394; Controlled for random effects of families; The *p*-values are obtained from a two-sided *z*-test; $^{***}p < .001$, $^{**}p < .01$, $^{*}p < .05$, $^{\bullet}p < .1$

Supplementary Table 24: Robustness Check for Table 2 including dyadic embeddedness and tie strength with important tie

Dependent Variable = Time until first outbound call to non-family plan member (hours)	Coef.	S.D.	Lower 2.5% CI	Upper 97.5% CI	N_eff	Rhat
(1) Ego and family all unembedded	-30.300	4.862	-40.127	-20.882	18359	1.000
(2) Ego and family all embedded	68.534	4.544	59.677	77.350	14300	1.000
Earthquake intensity group (1 = severe)	-15.582	4.533	-24.413	-6.727	15401	1.000
(1)*earthquake intensity group	-16.390	5.138	-26.618	-6.121	14954	1.000
(2)*earthquake intensity group	16.669	4.712	7.349	26.037	15067	1.000
ln(Degree centrality of ego)	-68.620	4.340	-76.980	-59.905	11911	1.000
ln(Total call frequency of ego)	-56.003	3.594	-63.085	-48.949	10133	1.000
ln(Total text frequency of ego)	-26.170	2.812	-31.685	-20.625	12587	1.000
ln(Internet usage frequency of ego)	1.997	2.529	-2.979	6.973	11414	1.000
ln(Phone retail price in Yuan)	35.716	2.672	30.461	41.002	9731	1.000
ln(Total Wechat frequency)	-7.436	3.034	-13.367	-1.531	12637	1.000
ln(Total frequency of other instant messaging)	-13.495	2.721	-18.821	-8.084	10539	1.000
Roaming dummy (1 = traveling outside of prefecture)	26.171	4.793	16.838	35.554	18109	1.000
Rural dummy (1 = rural)	21.499	4.435	12.768	30.071	15591	1.000
Damage dummy (1 = cell towers damaged in neighborhood)	-1.421	4.940	-11.209	8.271	14668	1.000
Prior intra-family callout share	28.984	4.964	19.246	38.881	15356	1.000
Dyadic embeddedness with important tie	126.818	2.966	121.025	132.635	11855	1.000
Tie strength with important tie	138.834	3.003	133.010	144.767	14861	1.000
(Intercept)	12.418	4.896	2.867	22.098	13846	1.000

Notes. Number of obs. = 18,958; Controlled for random effects of families. Bayesian estimations produce credible intervals (CI), which are the intervals within which the parameter values fall at some particular probability.

Triadic motifs and changes in intra-family communications

We also examined how triadic communications differed for families with different embeddedness structures. Here, we captured intra-family communications patterns with motif structures. Note that the edges of these motifs denote whether or not two nodes (i.e., family members) have had direct communications. There are 16 unique types of possible motifs (Harary et al. 1965) of mobile communications within traidic family that can be classified into three groups: one edge motifs (i.e., 2 and 3), two edge motifs (i.e., 4-9), and three edge motifs (10-16). The motif 1 is null and can be a reference in our analysis.

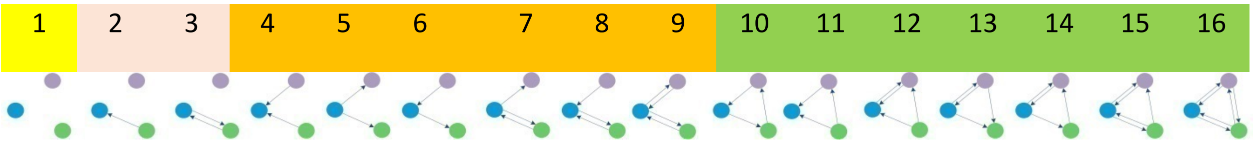

According to structural balance (Heider 1946) and clusterability (Davis 1967), motifs 3 and 16 are balanced, and motifs 1, 3, and 16 are clustered. Transitivity (Holland & Leinhardt 1970) is defined by 'friend of a friend is a friend' rule (Motifs 1-5, 11-13, and 16); non-vacuous transitivity adds the necessary condition that an i-j-k path exists (Motifs 11-13, and 16).

The mobile communications data and natural experimental context of the current study provides an unique test of structual belance and transitivity. We calculated a weekly transition matrix of motifs befor and after earthquake, illustrated by Supplementary Figure 13. As summarized in Supplementary Table 25, we find that only 32.06% of motifs were balanced, 44.07% were clustered, and 61.27% were consistent with transitivity before earthquake; the corresponding percentages changed to 36.42%, 45.41%, and 58.41% respectively after earthquake. Thus, the violations of structual belance and clusterability appear commonplace while transitivity principles are also poor predictors of the patterns of communications within triadic families.

The transitivity of motifs may be affected by triadic embeddedness. Completely unembedded motifs are more transitive than fully embedded ones, and 69.76% (64.52%) of the former and only 49.42% (51.67%) of the latter were transitive before (after) earthquake. To compare how different embeddedness structures affected communications motif, we normalized the distributions of motif patterns before and after earthquake (by percentage values) for each of the four types of embeddedness structures (Fig 1C). When a triadic family is completely unembedded, 20.79% (15.35%) of families exhibit motif 1 (no communication among three members), while 4.18% (9.74%) of families exhibit motif 16 (Reciprocal communications among all three members) before (after) the earthquake. When triadic family is fully embedded, the proportion of motif 1 falls to 4.57% (3.61%) while motif 16 increases to 17.45% (24.13%) before (after) the earthquake (Supplementary Figure 14).

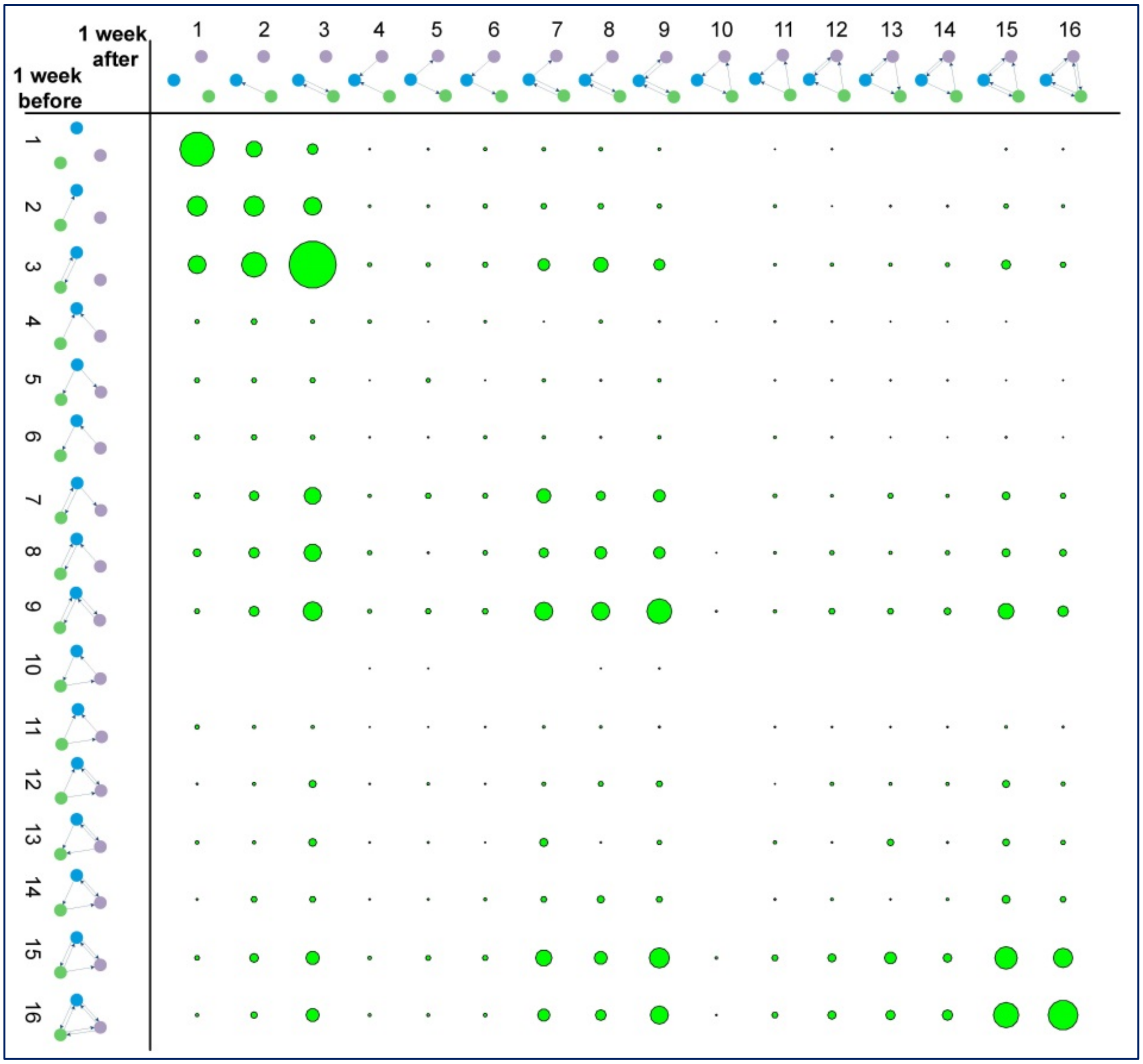

Supplementary Figure 13. Visualization of motif transition matrix before and after earthquake: All data. In the main text, Figure 3 shows change in triadic motifs before and after the earthquake for completely unembedded versus embedded family structures. Supplementary Figure 13 shows change in triadic motifs for all data (i.e., all family structures).

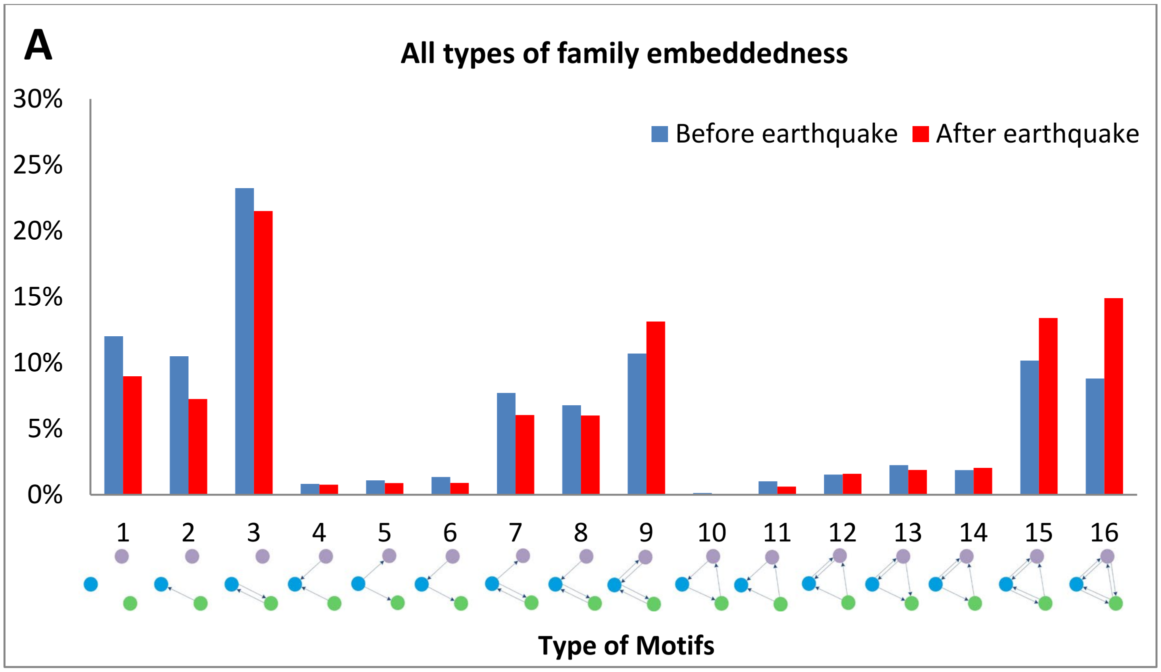
A
All types of family embeddedness
30%
25%
20%
15%
10%
5%
0%
Before earthquake
After earthquake
1 2 3 4 5 6 7 8 9 10 11 12 13 14 15 16
Type of Motifs

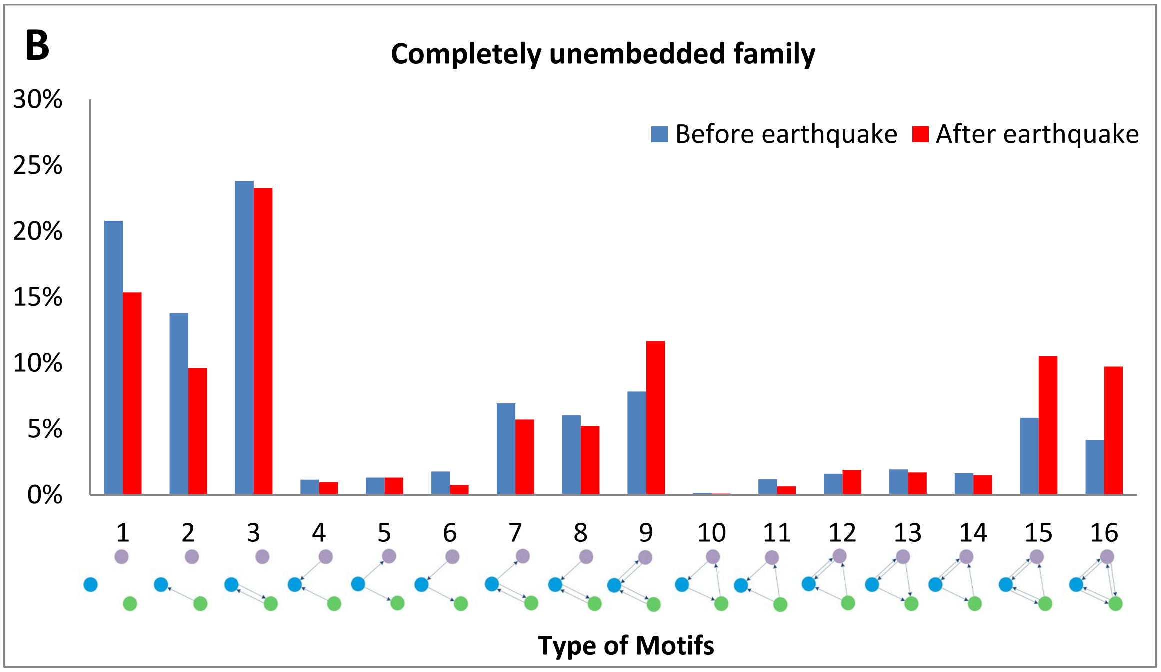
B
Completely unembedded family
30%
25%
20%
15%
10%
5%
0%
Before earthquake
After earthquake
1 2 3 4 5 6 7 8 9 10 11 12 13 14 15 16
Type of Motifs

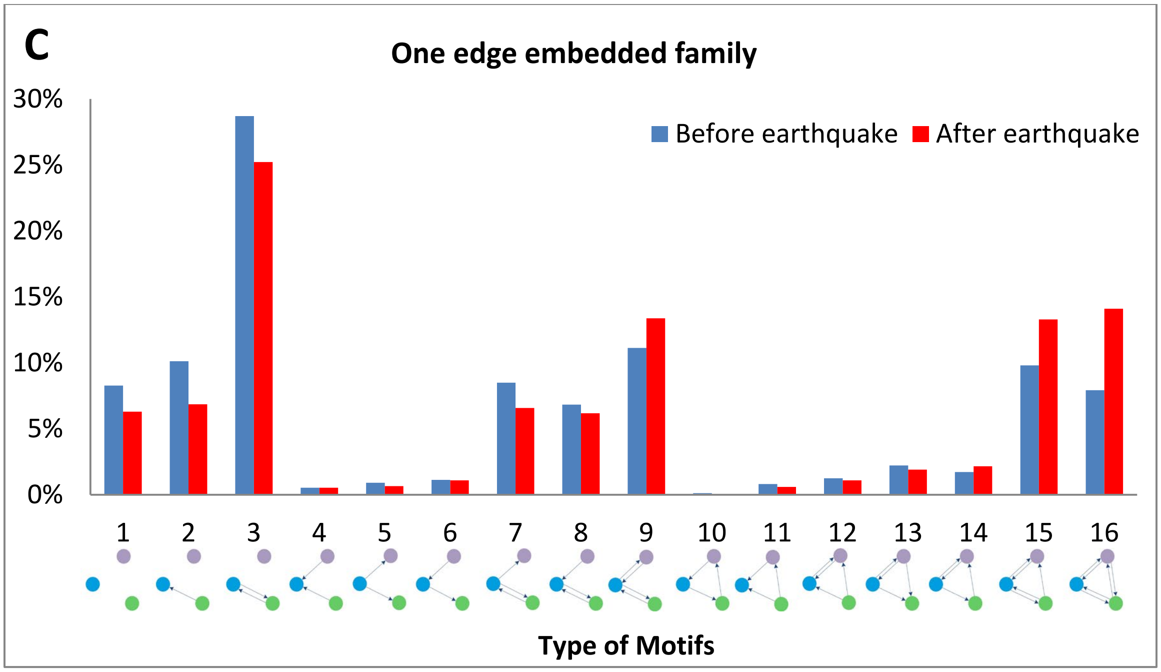
C
One edge embedded family
30%
25%
20%
15%
10%
5%
0%
Before earthquake
After earthquake
1 2 3 4 5 6 7 8 9 10 11 12 13 14 15 16
Type of Motifs

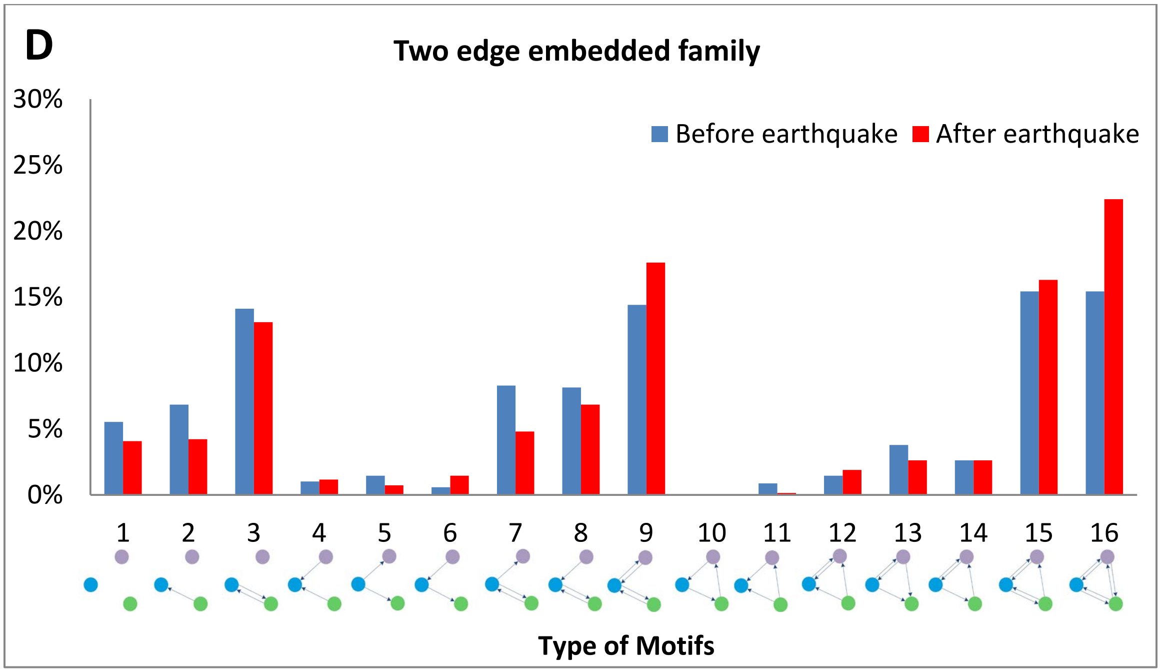
D
Two edge embedded family
30%
25%
20%
15%
10%
5%
0%
Before earthquake
After earthquake
1 2 3 4 5 6 7 8 9 10 11 12 13 14 15 16
Type of Motifs

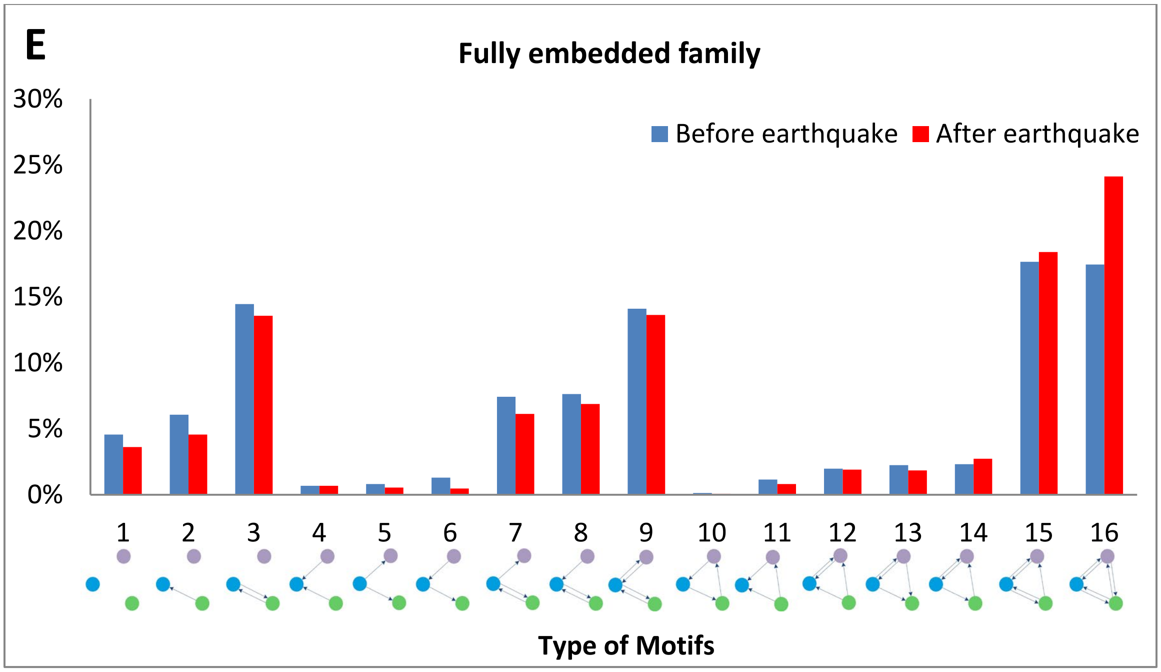

Supplementary Figure 14. Relative change of motifs before and after earthquake for different embeddedness structures (A-E). Supplementary Figures 14B (fully unembedded family) and E (fully embedded family) are alternative visualizations of Figure 4 in the main text, and illustrate that motifs with more reciprocal edges are relatively more frequent for fully embedded families (E). One- and two- edge embedded families show patterns intermediate to fully unembedded and fully embedded families.

Supplementary Table 25. Counts for types of motifs with following social network properties

All data

	Before earthquake	After earthquake
Balance	32.1%	36.4%
Cluster	44.1%	45.4%
Transitivity	61.3%	58.4%
Non-vacuous transitive	13.6%	19.0%

Embed = 0

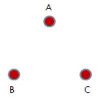

	Before earthquake	After earthquake
Balance	28.0%	33.0%
Cluster	48.8%	48.4%
Transitivity	69.8%	64.5%
Non-vacuous transitive	8.9%	14.0%

Embed = 1

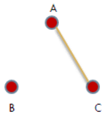

	Before earthquake	After earthquake
Balance	36.6%	39.3%
Cluster	44.9%	45.6%
Transitivity	60.8%	57.3%
Non-vacuous transitive	12.2%	17.7%

Embed = 2

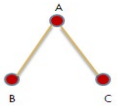

	Before earthquake	After earthquake
Balance	29.5%	35.5%
Cluster	35.1%	39.6%
Transitivity	50.5%	50.4%
Non-vacuous transitive	21.5%	27.1%

Embed = 3

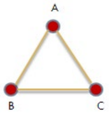

	Before earthquake	After earthquake
Balance	31.9%	37.7%
Cluster	36.5%	41.3%
Transitivity	49.4%	51.7%
Non-vacuous transitive	22.8%	28.7%

Macro-network effects and giant connected component graphs

Overall, we found that three-person networks rapidly form a giant connected component after the earthquake (Supplementary Fig. 16-17). However, some clusters had relatively fewer inter-family communications (Supplementary Fig. 18), while other clusters had relatively more inter-family calls (Supplementary Fig. 19). Overall, although intra-family calls were the dominant form of communications (363,184 calls, a 30.7% increase), there was also greater relative increase in inter-family calls (86,918 calls after the earthquake, a 49.2% increase from previous week). The former effect may be partly driven by greater initial focus and social support from within families with more fully embedded structures; Model 4 suggests that the latter effect may also be driven by families with more fully embedded structures.

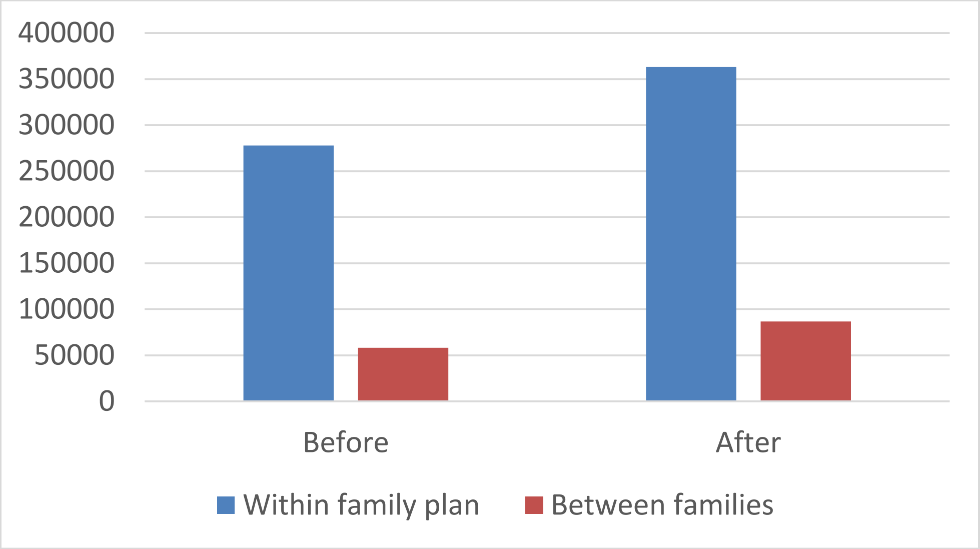

Supplementary Figure 15. Intra vs. extra- family communications after earthquake. Supplementary Figure 15 shows that there was a greater relative increase in inter-family calls (86,918 calls after the earthquake, a 49.2% increase from previous week), intra-family calls were the dominant form of communications (363,184 calls, a 30.7% increase).

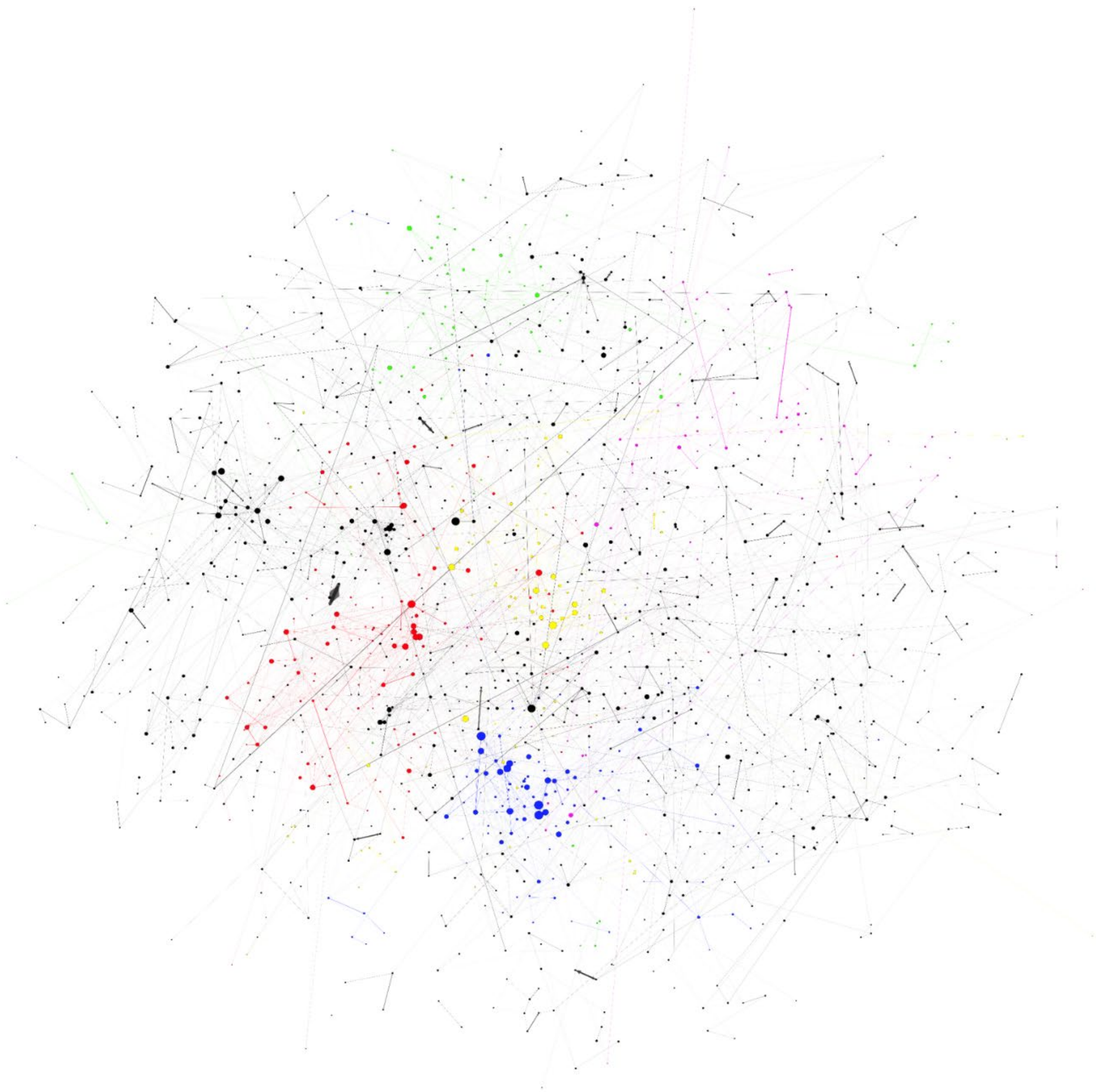

Supplementary Figure 16. Communications within and between families before the earthquake.

Supplementary Figure 16 shows 3-person families connected within the Giant Connected Component before the earthquake. Each node is a 3-person family. Node size corresponds to frequency of within-node communications, edge-width corresponds to frequency of between-node communications. Coloring based on clustering (6 total).

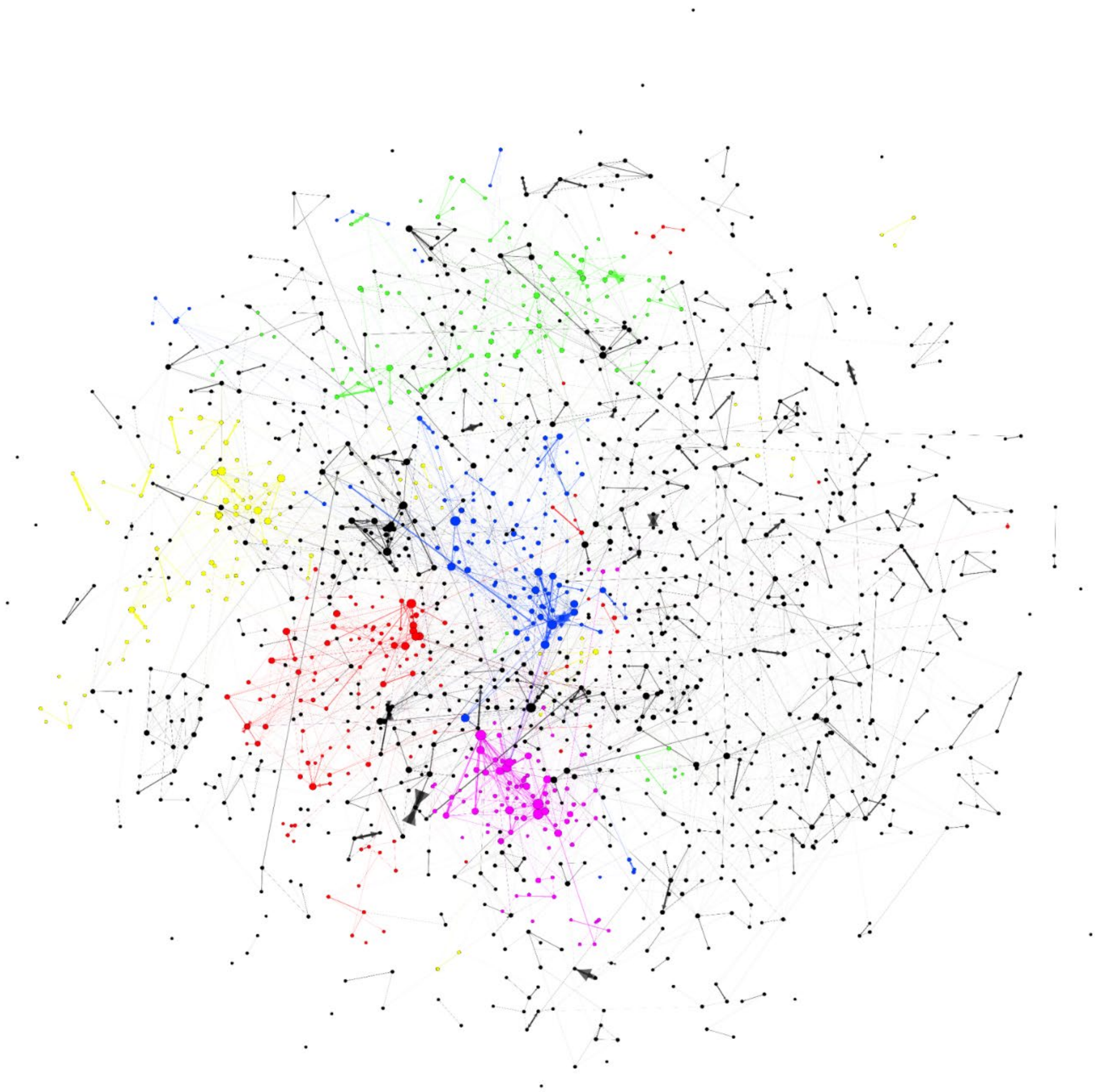

Supplementary Figure 17. Communications within and between families after the earthquake.

Supplementary Figure 17 shows 3-person families connected within the Giant Connected Component after the earthquake. Each node is a 3-person family. Node size corresponds to frequency of within-node communications, edge-width corresponds to frequency of between-node communications. Colouring based on clustering (6 total).

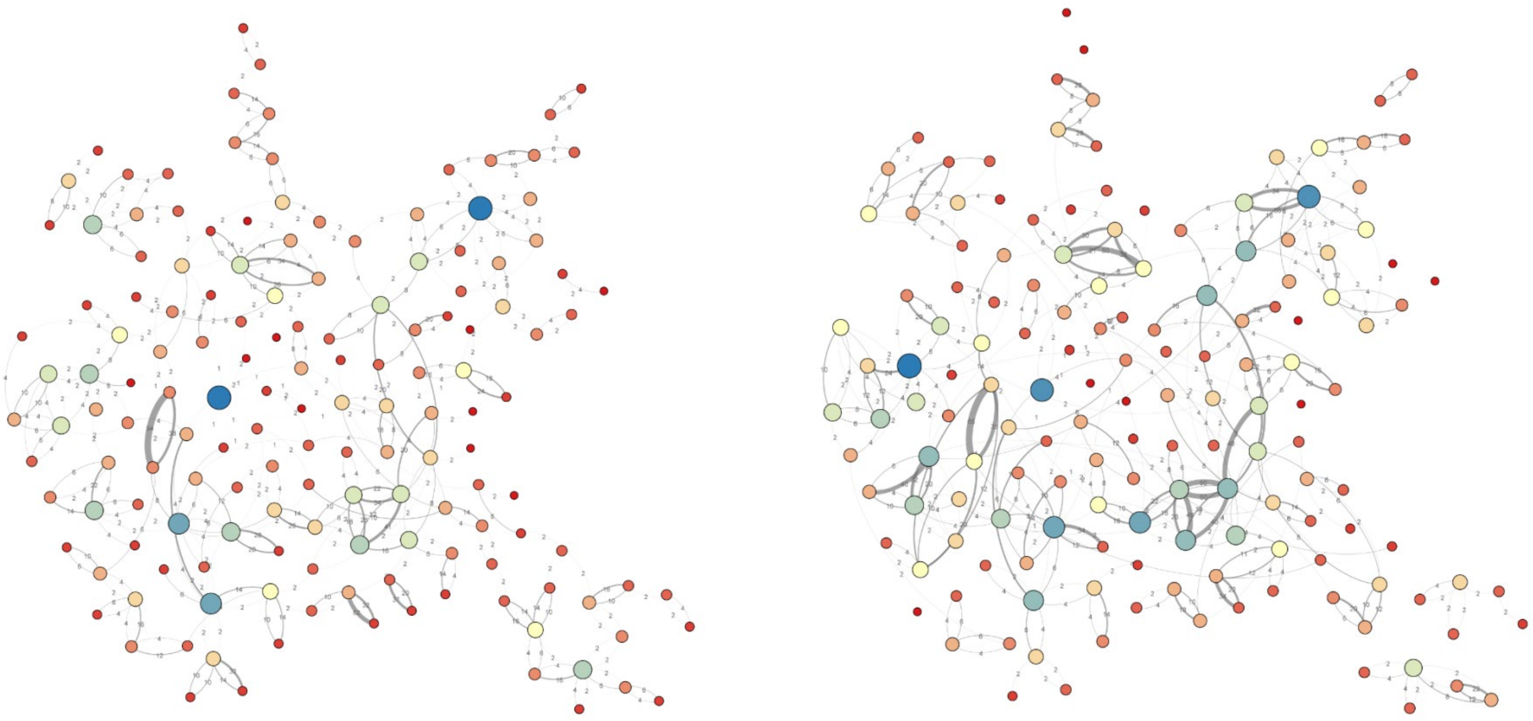

Supplementary Figure 18. **A cluster with relatively fewer inter-family communications before (left) and after (right) earthquake.**

Supplementary Figure 18 shows a specific cluster of the Giant Connected Component in Supplementary Figure 17.

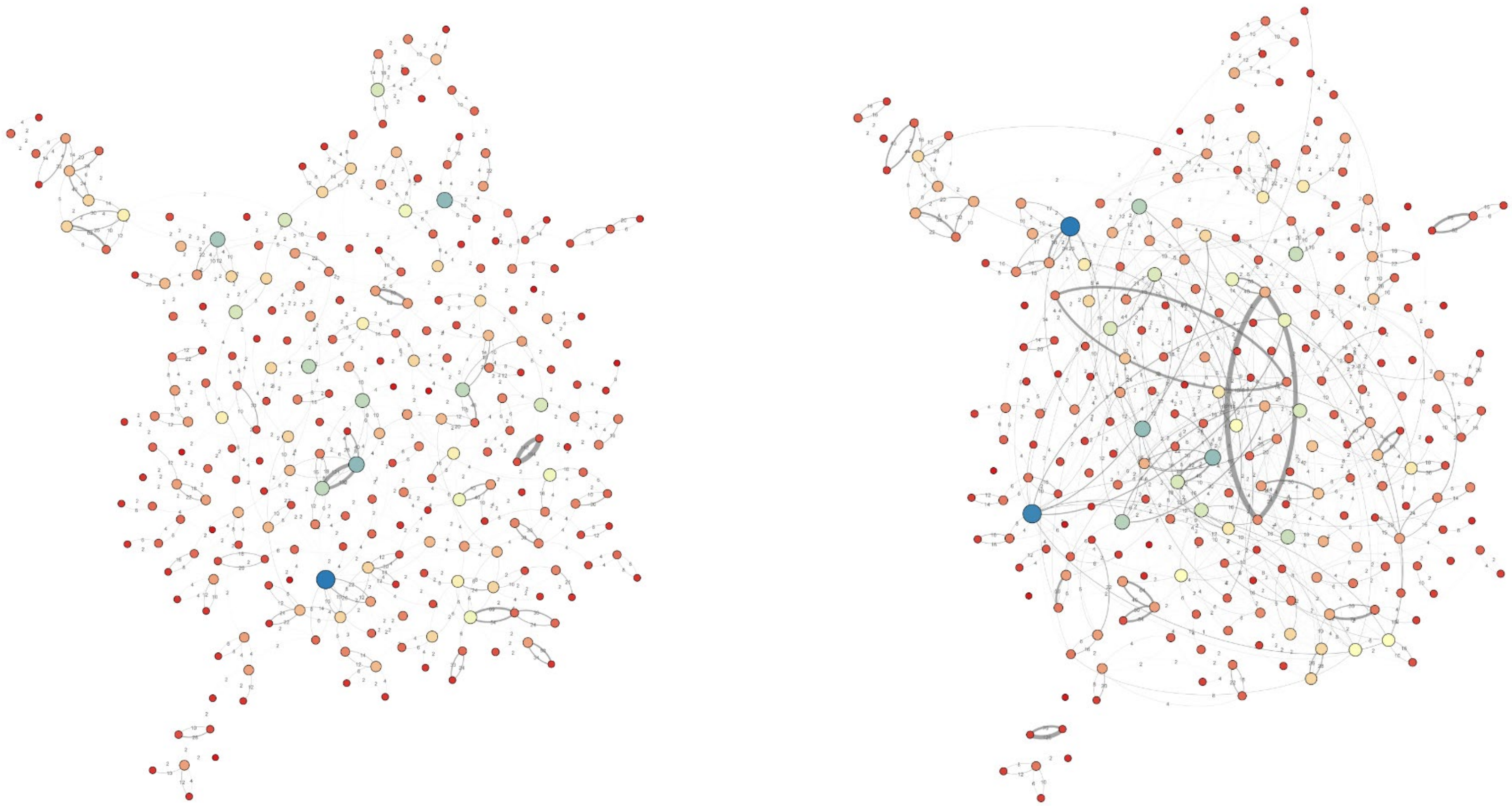

Supplementary Figure 19. A cluster with relatively more inter-family communications before (left) and after (right) earthquake.

Supplementary Figure 19 shows a specific cluster of the Giant Connected Component in Supplementary Figure 17.

Supplementary References

1. Nowak, M. A. Five rules for the evolution of cooperation. *Science* **314**, 1560–1563 (2006).
2. Butzer, K. W. Collapse, environment, and society. *Proc Natl Acad Sci USA* **109**, 3632–3639 (2012).
3. International Federation of Red Cross and Red Crescent Societies. *World Disasters Report 2014* – data. Retrieved from http://www.ifrc.org/world-disasters-report-2014/data (2014).
4. Jia, J. S., Lu, X., Yuan, Y., Xu, G., Jia, J., & Christakis, N. A. Population flow drives spatio-temporal distribution of COVID-19 in China. *Nature* **582**, 389–394 (2020).
5. Lu, X., Bengtsson, L., Holme, P. Predictability of population displacement after the 2010 Haiti earthquake. *Proc Natl Acad Sci USA* **109**, 11576-11581(2012).
6. Phan, T. Q., Airoldi, E. M. A Natural Experiment of Social Network Formation and Dynamics. *Proc Natl Acad Sci USA* **112**, 6595-6600 (2015).
7. Blumenstock, J. E, Eagle, N., Fafchamps, M. Airtime transfers and mobile communications: Evidence in the aftermath of natural disasters. *J Dev Econ* **120**, 157-181(2016).
8. Bagrow, J. P., Wang, D., Barabasi, A.-L. Collective response of human populations to large scale emergencies. *PLoS ONE* **6**, e17680 (2011).
9. Gao, L., Song, C., Gao, Z., Barabási, A.-L., Bagrow, J. P., Wang, D. Quantifying information flow during emergencies, *Sci Rep* **4**, 3997 (2014).
10. Stern, M. J., Fullerton, A. S. The Network Structure of Local and Extra□Local Voluntary Participation: The Role of Core Social Networks. *Soc Sci Quart*, **90**, 553-575 (2009).
11. Burt, R. S. Decay functions. *Soc Networks* **22**, 1–28 (2000).
12. Roberts, S. B. G., Dunbar, R. I. M. Communication in social networks: effects of kinship, network size and emotional closeness. *Pers Relatsh* **18**, 439–45 (2011).
13. Shor, E., Roefls, D. J., Yogev, T. The strength of family ties: a meta-analysis and meta-regression of self-reported social support and mortality. *Social Network* **35**, 626–638 (2013).
14. Wenger, G. C. The special role of friends and neighbors. *J Aging Stud* **4**, 149–169 (1990).
15. Lee, G. R., Ishii-Kuntz, M. Social interaction, loneliness and emotional well-being among the elderly. *Res Aging* **1**, 117–126 (1987).
16. Granovetter, M. The strength of weak ties. *Am J Sociol* **78**, 1360–1380 (1973).
17. Granovetter, M. Economic action and social structure: the problem of embeddedness. *Am J Sociol* **91**, 481–510 (1985).
18. Burt, R. S. *Structural Holes*. Cambridge, Mass.: Harvard University Press (1992).
19. Easly, D., Kleinberg, J. *Networks, Crowds, and Markets*. Cambridge University Press, Cambridge UK (2010).
20. Uzzi, B. Social Structure and Competition in Interfirm Networks: The Paradox of Embeddedness. *Admin Sci Quart* **42**, 35-67 (1997).

21. Feld, S. Structural Embeddedness and Stability of Interpersonal Relations. *Soc Networks* **19**, 91- 95 (1997).
22. Bott, E. Family and Social Network (2nd ed.). New York. Free Press (1971).
23. Kim, S., Skvoretz J. Embedded trade: a third-party effect. *Soc Sci Quart* **91**, 964-983 (2010).
24. Jackson, M. O., Rogers, B. W., Zenou, Y. The Economic Consequences of Social-Network Structure. *J Econ Lit* **55**, 49-95 (2017).
25. Aral, S., Walker, D. Tie Strength, Embeddedness, and Social Influence: A Large-scale networked experiment. *Manage Sci* **60**, 1352-1370 (2014).
26. Holland, P., Leinhardt, S. A Method for Detecting Structure in Sociometric Data. *Am J Sociol* **76**, 492-513 (1970).
27. Holland, P., Leinhardt, S. Local Structure in Social Networks. *Sociol Methodol 7*, 1-45 (1976).
28. Harary F., Norman, R. Z., Cartwright D. Structural Models: An Introduction to the Theory of Directed Graphs. Wiley, New York (1965).
29. Heider, F. Attitudes and cognitive organization. Journal of Psychology **21**, 107-112 (1946).
30. Cartwright, D., Harary F. Structural balance: a generalization of Heiders theory. *Psychol Rev* **63**, 277-293 (1956).
31. Milo, R. et al. Network motifs: Simple building blocks of complex networks. *Science* **298**, 824–827 (2002).
32. Milo, R. et al. Super families of evolved and designed networks. *Science* **303**, 1538–1542 (2004).
33. Kovanen, L., Kaski, K., Kertész, J., Saramäki, J. Temporal motifs reveal homophily, gender-specific patterns, and group talk in call sequences. *Proc Natl Acad Sci USA* **110**, 18070-18075 (2013).
34. Benson, A., Abebe, R., Schaub, M., Jadbabaie, A., Kleinberg, J. Simplicial Closure and Higher-order Link Prediction. *Proc Natl Acad Sci USA* **115**, 11221-11230 (2018)
35. Lambiotte, R., Rosvall, M., Scholtes, I. From networks to optimal higher-order models of complex systems. *Nat Phys* **15**, 313–320 (2019)
36. Milardo, R. M., Allan, G. Social networks and marital relationships. In R. Milardo & S. Duck (Eds.), *Families as relationships* (pp. 117-133). London: Wiley (2000).
37. Rand, D., Nowak, M., Fowler, J., Christakis, N. Static network structure can stabilize human cooperation. *Proc Natl Acad Sci USA* **111**, 17093-17098 (2014).
38. Procidano, M. E., Heller, K. Measures of perceived social support from friends and from family: three validation studies. *Am J Commun Pyschol* **11**, 1–24 (1983).
39. Eagle, N., Pentland, A., Lazer, D. Inferring friendship network structure using mobile phone data. *Proc Natl Acad Sci USA* **106**, 15274-15278 (2009).

40. Saramäkia, J., Leichtb, E. A., Lópezb, E., Roberts, S. G. B., Reed-Tsochasb, F., Dunbar, R. I. M. Persistence of social signatures in human communication. *Proc Natl Acad Sci USA* **111**, 942–947 (2014).
41. Szell, M., Lambiotte, R., Thurner, S. Multirelational organization of large-scale social networks in an online world. *Proc Natl Acad Sci USA* **107**, 13636-13641 (2010).
42. Facchetti, G., Iacono, G., Altafini, C. Computing global structural balance in large-scale signed social networks. *Proc Natl Acad Sci USA* **108**, 20953-20958 (2011).
43. Isakov, A., Fowler, J. H., Airoldi, E. M., & Christakis, N. A. The Structure of Negative Social Ties in Rural Village Networks. *Sociol Sci* **6**, 197-218 (2019).
44. Jia, J. S., Jia, J., Hsee, C. K., & Shiv, B. The role of hedonic behavior in reducing perceived risk: evidence from postearthquake mobile-app data. *Psychol Sci* **28**, 23-35 (2017).
45. Onnela, J.P. et al. Structure and tie strengths in mobile communication networks. *Proc Natl Acad Sci USA* **104**(18): 7332-7336 (2007).
46. McPherson, M., Smith-Lovin, L., Brashears, M. E. Models and marginals: Using survey evidence to study social networks. *Am Sociol Rev* **74**, 670-681 (2009).
47. Gelfand, A. E., Smith, A. F., Lee, T. M. Bayesian analysis of constrained parameter and truncated data problems using Gibbs sampling. *J Am Stat Assoc* **87**, 523-532 (1992).
48. Barabási, A-L. The origin of bursts and heavy tails in human dynamics. *Nature* **435**: 207–211 (2005).
49. Jo, H.H., Saramäki, J., Dunbar, R.I.M., Kaski, K. Spatial patterns of close relationships across the lifespan. *Sci Rep* **4**: 6988 (2014).
50. Palchykov, V., Kaski, K., Kertesz, J., Barabasi, A-L., Dunbar RIM Sex differences in intimate relationships. *Sci Rep* **2**: 370 (2012).
51. Jones, J.J., Settle, J.E., Bond, R.M., Fariss, C.J., Marlow, C., Fowler, J.H. Inferring tie strength from online directed behavior. *PLoS ONE* **8**(1): e52168 (2013).
52. Croissant, Y., Zeileis, A., Croissant, M.Y. Package 'truncreg' (2016).
53. Freilich, M. The natural triad in kinship and complex systems. *Am Sociol Rev* 529-540 (1964).
54. Henningsen, A. Estimating censored regression models in R using the censReg Package. R package vignettes (2010).
55. Sweetser, D.A. Path consistency in directed graphs and social structure. *Am J Sociol* 73: 287-293 (1967).
