# Supplementary material for: Triadic embeddedness structure in family networks predicts mobile communication response to a sudden natural disaster
Source: Nat Commun. 2021 Jul 13;12:4286. doi: 10.1038/s41467-021-24606-7 (PMC8277904; doi:10.1038/s41467-021-24606-7)
Supplement: Supplementary file 3 — Reporting Summary [file 41467_2021_24606_MOESM3_ESM.pdf]

Corresponding author(s): Jianmin Jia, Jayson S. Jia

Last updated by author(s): Apr 18, 2021

Reporting Summary

Nature Research wishes to improve the reproducibility of the work that we publish. This form provides structure for consistency and transparency in reporting. For further information on Nature Research policies, see our Editorial Policies and the Editorial Policy Checklist.

Statistics

For all statistical analyses, confirm that the following items are present in the figure legend, table legend, main text, or Methods section.

n/a	Confirmed	
☐	☒	The exact sample size (*n*) for each experimental group/condition, given as a discrete number and unit of measurement
☐	☒	A statement on whether measurements were taken from distinct samples or whether the same sample was measured repeatedly
☐	☒	The statistical test(s) used AND whether they are one- or two-sided<br>*Only common tests should be described solely by name; describe more complex techniques in the Methods section.*
☐	☒	A description of all covariates tested
☐	☒	A description of any assumptions or corrections, such as tests of normality and adjustment for multiple comparisons
☐	☒	A full description of the statistical parameters including central tendency (e.g. means) or other basic estimates (e.g. regression coefficient) AND variation (e.g. standard deviation) or associated estimates of uncertainty (e.g. confidence intervals)
☐	☒	For null hypothesis testing, the test statistic (e.g. *F*, *t*, *r*) with confidence intervals, effect sizes, degrees of freedom and *P* value noted<br>*Give P values as exact values whenever suitable.*
☐	☒	For Bayesian analysis, information on the choice of priors and Markov chain Monte Carlo settings
☒	☐	For hierarchical and complex designs, identification of the appropriate level for tests and full reporting of outcomes
☒	☐	Estimates of effect sizes (e.g. Cohen's *d*, Pearson's *r*), indicating how they were calculated

Our web collection on statistics for biologists contains articles on many of the points above.

Software and code

Policy information about availability of computer code

Data collection	No software was used.
Data analysis	We have used open source software R (R version 3.5.1) to analyze the data in this study.

For manuscripts utilizing custom algorithms or software that are central to the research but not yet described in published literature, software must be made available to editors and reviewers. We strongly encourage code deposition in a community repository (e.g. GitHub). See the Nature Research guidelines for submitting code & software for further information.

Data

Policy information about availability of data

All manuscripts must include a data availability statement. This statement should provide the following information, where applicable:
- Accession codes, unique identifiers, or web links for publicly available datasets
- A list of figures that have associated raw data
- A description of any restrictions on data availability

Two anonymized samples of the data that supports the findings of this study are provided. Large-scale datasets analyzed in the current study are not publicly available due to the agreement with the telecom carrier.

April 2020

Field-specific reporting

Please select the one below that is the best fit for your research. If you are not sure, read the appropriate sections before making your selection.

☐ Life sciences ☒ Behavioural & social sciences ☐ Ecological, evolutionary & environmental sciences

For a reference copy of the document with all sections, see nature.com/documents/nr-reporting-summary-flat.pdf

Behavioural & social sciences study design

All studies must disclose on these points even when the disclosure is negative.

Study description	In this mixed-methods empirical study, we demonstrate that families' communications dynamics after sudden environmental shock (Ya'an earthquake) can be predicted by triadic embeddedness structure (in family networks), a structural metric we propose that reflects the number of dyads in a family triad that share social ties.
Research sample	We used 4 months of anonymized telecommunications records (March-June 2013) regarding 35,565 active subscribers (who were in 11,855 3-person family plans) of a major Chinese mobile telecommunications carrier, who were residing in the Ya'an region of Sichuan. The data included time-stamped records of individuals' voice calls, text messages (SMS), mobile Internet usage, mobility (tower access), demographics, and customer data (e.g., phone model, spending, family plan membership). The earthquake occurred at 08:02 on April 20th. Most communication variables were derived from March 2013 data. Physical presence in Ya'an was verified by mobile phone cell tower reception during the earthquake; we also identified roaming subscribers for robustness checks. We also check for the stability of the call data between Jan. 2013 and June 2016, and whether WeChat had an effect (it did not) on our data in the SI (S2-3).
Sampling strategy	No sample-size calculation was performed, and thus sample size was determined by the existing anonymized telecommunications records. The sample contains all records of the 3-person family plans and was the largest existing sample that a major Chinese mobile telecommunications carrier can provide.
Data collection	Data was recorded and collected by a major Chinese mobile telecommunications carrier, and the researchers were not involved in the data collection process.
Timing	The data collection starts in April 2016 and ends in May 2016. There were no gap in or between the collection periods.
Data exclusions	No data were excluded from the analyses.
Non-participation	No participants dropped out/declined participation.
Randomization	Our study is an empirical case study and random assignments of participants were not performed.

Reporting for specific materials, systems and methods

We require information from authors about some types of materials, experimental systems and methods used in many studies. Here, indicate whether each material, system or method listed is relevant to your study. If you are not sure if a list item applies to your research, read the appropriate section before selecting a response.

Materials & experimental systems

n/a	Involved in the study	
☒	☐	Antibodies
☒	☐	Eukaryotic cell lines
☒	☐	Palaeontology and archaeology
☒	☐	Animals and other organisms
☐	☒	Human research participants
☒	☐	Clinical data
☒	☐	Dual use research of concern

Methods

n/a	Involved in the study	
☒	☐	ChIP-seq
☒	☐	Flow cytometry
☒	☐	MRI-based neuroimaging

Human research participants

Policy information about studies involving human research participants

Population characteristics	See Above.
Recruitment	Data analysis are conducted based on data the telecom carrier already collected, which was covered by their user agreement (that included use of anonymzied data by third parties for research purposes). There was no recruitment procedure involved and potential self-selection bias are minimized.

Ethics oversight	Ethical approval to use anonymized individual-level telecom data for academic research is granted by the Human Research Ethics Committee of The University of Hong Kong (EA1912107).

Note that full information on the approval of the study protocol must also be provided in the manuscript.
